# Abnormalities of brain structure and function in cervical spondylosis: a multi-modal voxel-based meta-analysis

**DOI:** 10.3389/fnins.2024.1415411

**Published:** 2024-06-14

**Authors:** Lulu Cheng, Jianxin Zhang, Hongyu Xi, Mengting Li, Su Hu, Wenting Yuan, Peng Wang, Lanfen Chen, Linlin Zhan, Xize Jia

**Affiliations:** ^1^School of Foreign Studies, China University of Petroleum (East China), Qingdao, China; ^2^Shanghai Center for Research in English Language Education, Shanghai International Studies University, Shanghai, China; ^3^School of Western Studies, Heilongjiang University, Harbin, China; ^4^School of Psychology, Zhejiang Normal University, Jinhua, China; ^5^Key Laboratory of Intelligent Education Technology and Application of Zhejiang Province, Zhejiang Normal University, Jinhua, China; ^6^English Department, Heilongjiang International University, Harbin, China; ^7^Department of Language, Literature and Communication, Faculty of Humanities, Vrije Universiteit Amsterdam, Amsterdam, Netherlands; ^8^Department of Psychology, Education, and Child Studies, Erasmus School of Social and Behavioural Sciences, Erasmus University Rotterdam, Rotterdam, Netherlands; ^9^School of Medical Imaging, Shandong Second Medical University, Weifang, Shandong, China

**Keywords:** amplitude of low-frequency fluctuations, cervical spondylosis, diffusion tensor imaging, meta-analysis, regional homogeneity, surface-based morphometry, voxel-based morphometry

## Abstract

**Background:**

Previous neuroimaging studies have revealed structural and functional brain abnormalities in patients with cervical spondylosis (CS). However, the results are divergent and inconsistent. Therefore, the present study conducted a multi-modal meta-analysis to investigate the consistent structural and functional brain alterations in CS patients.

**Methods:**

A comprehensive literature search was conducted in five databases to retrieve relevant resting-state functional magnetic resonance imaging (rs-fMRI), structural MRI and diffusion tensor imaging (DTI) studies that measured brain functional and structural differences between CS patients and healthy controls (HCs). Separate and multimodal meta-analyses were implemented, respectively, by employing Anisotropic Effect-size Signed Differential Mapping software.

**Results:**

13 rs-fMRI studies that used regional homogeneity, amplitude of low-frequency fluctuations (ALFF) and fractional ALFF, seven voxel-based morphometry (VBM) studies and one DTI study were finally included in the present research. However, no studies on surface-based morphometry (SBM) analysis were included in this research. Due to the insufficient number of SBM and DTI studies, only rs-fMRI and VBM meta-analyses were conducted. The results of rs-fMRI meta-analysis showed that compared to HCs, CS patients demonstrated decreased regional spontaneous brain activities in the right lingual gyrus, right middle temporal gyrus (MTG), left inferior parietal gyrus and right postcentral gyrus (PoCG), while increased activities in the right medial superior frontal gyrus, bilateral middle frontal gyrus and right precuneus. VBM meta-analysis detected increased GMV in the right superior temporal gyrus (STG) and right paracentral lobule (PCL), while decreased GMV in the left supplementary motor area and left MTG in CS patients. The multi-modal meta-analysis revealed increased GMV together with decreased regional spontaneous brain activity in the left PoCG, right STG and PCL among CS patients.

**Conclusion:**

This meta-analysis revealed that compared to HCs, CS patients had significant alterations in GMV and regional spontaneous brain activity. The altered brain regions mainly included the primary visual cortex, the default mode network and the sensorimotor area, which may be associated with CS patients' symptoms of sensory deficits, blurred vision, cognitive impairment and motor dysfunction. The findings may contribute to understanding the underlying pathophysiology of brain dysfunction and provide references for early diagnosis and treatment of CS.

**Systematic review registration:**

https://www.crd.york.ac.uk/PROSPERO/, CRD42022370967.

## 1 Introduction

Cervical spondylosis (CS) is a common degenerative condition of the cervical spine that predominantly affects middle-aged and elderly populations (Reddy et al., 2019). The disorder may develop into different conditions depending on the location of nerve compression and the stage of the disease development (Takagi et al., 2011). For example, pure spondylosis was usually accompanied by axial neck pain or stiffness (Takagi et al., 2011), cervical radiculopathy was caused by compression of spinal nerve and showed symptoms of shooting or burning pain in the neck, paresthesia or motor weakness related to the disordered nerve root (Shedid and Benzel, 2007; Theodore, 2020), while cervical myelopathy was generated by compression of spinal cord due to the deterioration of cervical spine, which may lead to neck pain or stiffness, weakness or numbness in the upper and/or lower extremity (Takagi et al., 2011; Kalsi-Ryan et al., 2013; Theodore, 2020). Studies have found that people may suffer from one or several types of CS such as both cervical radiculopathy and myelopathy (Yu et al., 1986), all of which have significant impacts on our daily life (Badhiwala and Wilson, 2018). The symptoms of CS are mostly considered to be caused by cervical spine or spinal cord function injury, so previous studies on CS mainly focused on local lesions of cervical spine and spinal cord (Berberat et al., 2023). However, scholars have detected that the dysfunction of cervical spine or spinal cord alone could not explain the connections between CS-related symptoms, and cervical spinal decompression sometimes did not relieve the symptoms of some patients and even worsened the condition (Sun L. et al., 2016; Wu and Wang, 2023; Fard et al., 2024). On this basis, scholars began to explore related changes of the brain after the degeneration or injury of cervical spine and found that the brain function and structure of CS patients would undergo remodeling changes, which may then affect the clinical manifestations and prognosis of patients (Wu and Wang, 2023). However, the neurobiological mechanisms of CS remain unclear, indicating the need for further research to fully understand the disease.

Resting-state functional magnetic resonance imaging (rs-fMRI) is an effective tool for exploring the neural mechanisms of various diseases (Khan et al., 2024). It examines the spontaneous fluctuations in the blood oxygen level-dependent (BOLD) signal (Biswal et al., 1997). Among the analytical methods of rs-fMRI, functional connectivity (FC) (including region of interest and seed-based FC) examines the synchronicity or similarity of functional activities between remote brain regions through the calculation of the correlation of time series (Friston et al., 1993; Biswal et al., 1995). However, it could not identify the specific abnormal areas of the brain (Zang et al., 2007). To complementing this, the amplitude of low-frequency fluctuation (ALFF), fractional ALFF (fALFF) and regional homogeneity (ReHo) are well-established and widely-utilized for examining regional spontaneous brain activity (Yang et al., 2019; Wang et al., 2022; Chang et al., 2023). Specifically, ALFF and fALFF gauge the intensity of spontaneous brain activity within a single voxel during rest (Zou et al., 2008), whereas ReHo evaluates the synchronization of the BOLD signal across a focal voxel and its 26 surroundings (Zang et al., 2004). The integrative application of ALFF/fALFF and ReHo has been evidenced to provide more comprehensive complementary insights into regional spontaneous brain activity (Salvia et al., 2019; Yao et al., 2021). Besides, voxel-based morphometry (VBM) and surface-based morphometry (SBM) are effective approaches to measure the indexes of cortical morphology by using T1-weighted MRI scans (Goto et al., 2022). Specifically, VBM offers a standardized approach to assessing gray matter volume (GMV) (Whitwell, 2009), while SBM calculate such morphological characteristics as cortical thickness, surface area, sulcus depth, gyrification index and fractal dimension (Riccelli et al., 2017). Scholars also have found that VBM and SBM can be used as complementary methods to detect the morphological alterations of the gray matter, which can improve the accuracy of the detection results (Goto et al., 2022). In addition to the study of the function and structure of gray matter, research on white matter has also been paid more attention. Recently, diffusion tensor imaging (DTI) has become an effective means to investigate the microstructure of white matter beyond the structural dimensions evaluated by T1 and T2 weighted MRI (Qiu et al., 2015). Combining the structural and functional studies on gray matter, we can get a more comprehensive picture of gray matter (Dang et al., 2022), while the combination of studies on gray matter and white matter could advance our understanding of the cerebral microstructure.

Recently, a growing body of research has employed rs-fMRI, structural MRI and DTI to explore functional and structural brain anomalies in CS patients (Bernabéu-Sanz et al., 2020; Chang et al., 2023), which have advanced our knowledge of the pathophysiology of CS. However, the findings of previous neuroimaging research varied, leading to diverse and inconsistent evidence, and the persistent neurological alterations related with CS remain largely unknown. For example, previous studies on regional spontaneous brain activity in the middle frontal gyrus (MFG) in CS patients have produced mixed outcomes, with some finding hyperactivity, some finding hypoactivity while the other finding no abnormal change in this area (Xu et al., 2018; Yue and Du, 2020; Bai et al., 2022). Complex results were also found in structural neuroimaging studies on CS (Woodworth et al., 2019; Wang et al., 2023). The inconsistency among different studies could be due to small sample sizes, different data processing methods, publication bias toward positive results and flexible analytical methods (Tahmasian et al., 2019; Sun et al., 2020; Liu et al., 2021). To account for this, meta-analysis has emerged as an objective, effective and efficient method to integrate the findings of prior studies and identify more definitive brain regions that are persistently involved in the pathophysiology of a specific disorder, namely, to create the “collective mind” (Fox et al., 2014), thus enhancing sample size, statistical power, the reliability and replicability of findings (Radua and Mataix-Cols, 2012; Tahmasian et al., 2019). Recently, it has become increasingly popular in addressing discrepancies in clinical research and has been employed to investigate the persistent brain alterations in a variety of disease such as anxiety disorder, major depression and autism spectrum disorder (Serra-Blasco et al., 2021; Wang et al., 2022), but there is no systematic meta-analysis of neuroimaging studies related to cervical spondylosis. Although three systematic reviews on CS, which have been published recently, have provided an overview of CS-related neuroimaging studies from a macroscopic perspective, they did not examine the most consistent and core brain alterations in CS patients (Wu and Wang, 2023; Fard et al., 2024; Khan et al., 2024). However, the identification of the consistent and core brain alterations may help us understand its underlying neuropathological basis, further explain the symptoms of CS patients, and facilitate the diagnosis and treatment of the disease (Tahmasian et al., 2019). In this sense, it is of vital clinical and research significance to conduct a meta-analysis to reach a consistent conclusion.

Therefore, in the present study, we conducted a multi-modal voxel-based meta-analysis of rs-fMRI, structural MRI and DTI studies to investigate the most consistent brain alterations for each modality in CS patients, aiming to advance the understanding of CS pathogenesis. Given that different conditions of CS may occur simultaneously and share some key clinical manifestations such as neck pain, numbness or stiffness (Wang et al., 2016), we included studies on different stages of the disease, encompassing pure spondylosis, cervical radiculopathy and cervical myelopathy.

## 2 Methods

### 2.1 Data sources and study selection

This meta-analysis was conducted according to the Preferred Reporting Items for Systematic Reviews and Meta-Analyses (PRISMA) guidelines (Page et al., 2021) (Supplementary Tables S1, S2) and registered in the PROSPERO International Prospective Register of Systematic Reviews (register number: CRD42022370967) (https://www.crd.york.ac.uk/PROSPERO/). To collate relevant studies on brain structural and functional differences between CS and healthy controls (HCs), a comprehensive and systematic literature search was executed up until April 30, 2024 across five Chinese and English databases including Embase, PubMed, Web of Science, Chinese National Knowledge Infrastructure (CNKI) and Wanfang Data. The following keywords were employed to identify related rs-fMRI studies: (“cervical spondylosis” OR “CS” OR “CSD” OR “cervical spondylotic” OR “cervical radiculopathy” OR “CSR” OR “cervical myelopathy” OR “CSM” OR “DCM”) AND (“ReHo” OR “regional homogeneity” OR “amplitude of low-frequency fluctuations” OR “ALFF” OR “fractional amplitude of low-frequency fluctuations” OR “fALFF”), while the following ones were for VBM studies: (“cervical spondylosis” OR “cervical spondylotic” OR “cervical radiculopathy” OR “cervical myelopathy” OR “CS” OR “CSD” OR “CSM” OR “DCM”) AND (“voxel-based morphometry” OR “VBM” OR “voxel-wise” OR “voxel-based” OR “volumetric” OR “morphometry” OR “gray matter”) AND (“magnetic resonance imaging” OR “MRI” OR “neuroimaging”). As for relevant SBM studies, we used such keywords as (“cervical spondylosis” OR “cervical spondylotic” OR “cervical myelopathy” OR “cervical radiculopathy”) AND (“SBM” OR “surface-based morphometry” OR “cortical thickness” OR “surface area” OR “sulcus depth” OR “gyrification index” OR “fractal dimension”). Meanwhile, relevant DTI studies were retrieved by the following keywords: (“cervical spondylosis” OR “cervical spondylotic” OR “cervical myelopathy” OR “cervical radiculopathy”) AND (“DTI” OR “diffusion tensor imaging” OR “diffusion tensor magnetic resonance imaging” OR “diffusion tensor MRI” OR “diffusion tensor MRIs” OR “Diffusion Tractography”) AND (“brain” OR “cerebral” OR “cortex” OR “subcortex” OR “cortical” OR “subcortical” OR “cerebrum”). Detailed search strategies for each database were shown in Supplementary Table S3. To ensure an exhaustive coverage, the references of selected studies and review articles were also scrutinized.

Inclusion criteria were as follows: (1) Diagnosis of CS in patients, including pure spondylosis, cervical myelopathy, and cervical radiculopathy; (2) rs-fMRI studies using ReHo, ALFF, or fALFF analytical methods, structural MRI studies including VBM or SBM research, and relevant DTI studies; (3) Conducting brain imaging comparisons between CS and HCs; (4) Reporting whole-brain results in Montreal Neurological Institute (MNI) or Talairach coordinates (Radua and Mataix-Cols, 2009; Müller et al., 2018). Exclusion criteria included: (1) Non-empirical or nonhuman research such as review, conference abstract and animal research; (2) Studies only reporting region of interest results, which would bias the meta-analytic findings (Radua and Mataix-Cols, 2009; Müller et al., 2018); (3) Studies failing to report MNI or Talairach coordinates. Longitudinal or intervention studies were included only for their baseline data. Among studies with overlapping samples, preference was given to the study with the largest sample size and most comprehensive information (Zhao et al., 2023).

The quality of each included study was evaluated using a 20-point checklist in previous studies since it can reflect key variables that are significant for evaluating neuroimaging studies (Iwabuchi et al., 2015; Pan et al., 2017). Specifically, the checklist, as shown in Supplementary Table S4, contains two categories with 13 questions assessing the sample characteristics (e.g., the diagnostic criteria, demographic and clinical information on the study samples of the included study), methodology (e.g., neuroimaging acquisition parameters) and the quality of reporting results (e.g., statistical correction methods). Meanwhile, the characteristics of the included studies, including the diagnostic criteria, types of CS, the number, gender ratio and mean age of participants in both CS and HCs groups, analytical methods, statistical thresholds, research design, the peak coordinates of differential brain regions and their corresponding effect sizes were extracted from each included study. The process of literature search, selection, quality assessment and data extraction were independently executed by two authors. Discrepancies were resolved through consultation with a third author to reach consensus.

### 2.2 Voxel-wise meta-analyses of structural and functional alterations

Due to the insufficient number of SBM and DTI studies that could be included in this meta-analysis, we mainly detailed the meta-analysis of rs-fMRI and VBM studies in the methodological part. Individual meta-analysis was conducted to discern brain structural or functional differences using Anisotropic Effect-size Signed Differential Mapping (AES-SDM) software (version 5.15, https://www.sdmproject.com/software/). Specifically, for each meta-analysis, peak coordinates of statistically significant clusters between CS and HCs together with their corresponding effect sizes (*T*-values) were extracted from each included study and compiled into separate text files (Radua et al., 2014). Here, we included whole-brain analyses both with and without multiple comparison correction since according to Radua and Mataix-Cols (2009), the inclusion of analysis without multiple comparison correction would not bias the possibility to identify significant findings. An effect size map for each study was then generated using an anisotropic Gaussian kernel (Radua et al., 2014). Subsequently, a mean map was computed employing a random-effects model, accounting for sample size, intra-study variability, and inter-study heterogeneity (Radua and Mataix-Cols, 2012; Pan et al., 2017; Su et al., 2022). The threshold of *p* < 0.005, peak height *Z* > 1, and cluster extent > 10 voxels was selected to balance sensitivity and specificity against false positives (Radua and Mataix-Cols, 2009; Radua et al., 2012b; Pan et al., 2017; Wang et al., 2022). The results of abnormal brain areas among CS patients in each neuroimaging modality were finally presented in MNI coordinates.

### 2.3 Multi-modal meta-analysis

Based on the two probability maps (*P*_*F*_ and *P*_*V*_) generated by rs-fMRI and VBM meta-analyses, respectively, a multi-modal meta-analysis was conducted to explore the overlapping or conjunction of functional and structural brain alterations in CS patients (Long et al., 2023). The usual multimodal approach was to overlap the regions of statistical significance in the two modes (Nichols et al., 2005), in other words, to obtain the intersection of the two maps. However, this approach assumes that *p*-values were calculated without error, which may not be the case in neuroimaging data where different statistical means, such as permutations and randomizations, evaluating the same hypothesis generate significantly different *p*-values (Radua et al., 2013). Therefore, we conducted the multimodal meta-analysis according to the refined overlap approach (Radua et al., 2012a, 2013). Specifically, the two probability maps (*P*_*F*_ and *P*_*V*_) generated by unimodal meta-analysis were combined so that the *p*-values could be amalgamated to determine a union of changes in the two modes (*U*), with *U* estimated as *U* = *P*_*V*_ + *P*_*F*_ – *P*_*V*_ × *P*_*F*_ (Radua et al., 2012a, 2013). However, this statistic of *U* in its original form would be obviously conservative, so it was optimized by *P* = *U* + (1 – *U*) × ln (1 – *U*), thus, reducing the disequilibrium of false positive and negative rates (Radua et al., 2012a, 2013). Meanwhile, the threshold of *p* < 0.0025 was employed for multimodal meta-analysis since in the unimodal meta-analysis, we used the threshold of *p* < 0.005, as suggested in previous studies (Radua et al., 2012a, 2013).

### 2.4 Analyses of jackknife sensitivity, heterogeneity, and publication bias

To ascertain the reliability and stability of the findings, jackknife sensitivity analysis was performed for each meta-analysis. This entailed iterative statistical re-evaluation, excluding one different dataset each time (Radua and Mataix-Cols, 2009; Pan et al., 2017). A finding was deemed robust if brain regions remained significant across most combinations of studies (Radua and Mataix-Cols, 2009). Heterogeneity analyses were separately executed for each meta-analysis, using a random-effects model with *Q*-statistics to determine the presence of unexplained inter-study variance (Iwabuchi et al., 2015; Wang et al., 2022). A voxel threshold of *p* < 0.005 with a peak height *Z* > 1 and a cluster extent > 10 voxels was set for identifying significant heterogeneity (Radua et al., 2012b; Su et al., 2022). Additionally, Egger test was performed to assess publication bias, utilizing peak coordinates from clusters where significant differences were observed between CS and HCs (Ioannidis et al., 2014; Wang et al., 2022). A *p*-value < 0.05 in the Egger test was indicative of significant publication bias (Pan et al., 2017; Wang et al., 2018, 2022).

### 2.5 Subgroup analyses

Two subgroup analyses were conducted to examine the possible sources of heterogeneity that occurred in rs-fMRI meta-analysis and to assess the potential impact of different analytical methods in rs-fMRI data processing and different conditions of the disease. Specifically, the subgroup analyses of ALFF and ReHo were performed to analyze the effects of methods on abnormal brain areas. In addition, given that cervical myelopathy was caused by compression of spinal cord, a part of the central nervous system, which is different from the generation of pure spondylosis and cervical radiculopathy (Takagi et al., 2011; Wang et al., 2016; Theodore, 2020), a subgroup analysis of different types of the disease was performed to explore specific functional brain changes for different conditions, mainly including cervical myelopathy and CS without myelopathy. However, the subgroup analysis was not conducted in the VBM meta-analysis due to the small sample number of each subgroup.

### 2.6 Meta-regression analyses

Based on AES-SDM, general linear meta-regression of random effects was conducted to analyze the potential effects of related clinical and demographic characteristics on alterations in brain structure and function among CS patients. The independent variables of meta-regression include the Japanese Orthopedic Association (JOA) scores, the mean age and the percentage of female, while the dependent variable was the SDM values for meta-analysis of each mode. To reduce false positives, we set the significance threshold to *p* < 0.0005, *Z* > 1, and cluster size > 10 according to previous studies (Radua et al., 2012a; Yao et al., 2021; Cheng et al., 2023). Only those clusters demonstrating significant changes in one of the extremes of the regressor and slope were reported (Radua et al., 2012a). Meanwhile, the results that fall outside the main meta-analysis were discarded (Radua and Mataix-Cols, 2009; Radua et al., 2012a).

## 3 Results

### 3.1 Included studies and sample characteristics

**Figure 1:**
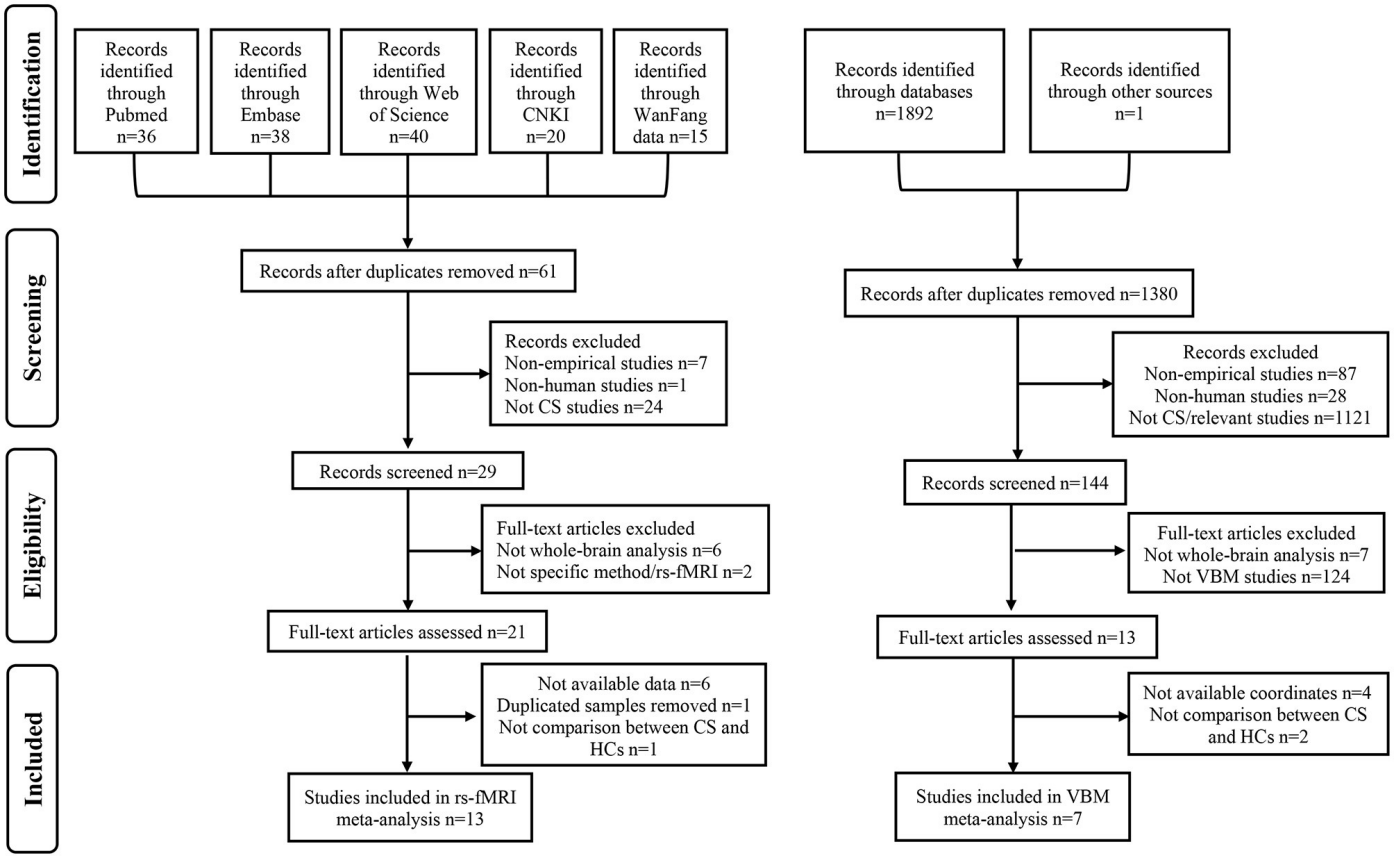
Flow diagram of literature search and study selection. CNKI, Chinese National Knowledge Infrastructure; CS, cervical spondylosis; rs-fMRI, resting-state functional magnetic resonance imaging; HCs, healthy controls; VBM, voxel-based morphometry.

A total of 21 studies were included in this meta-analysis (Table 1; Figure 1; Supplementary Figure S1): 13 rs-fMRI studies encompassing 486 CS patients (mean age ± SD: 46.21 ± 13.86 years) and 439 HCs (44.86 ± 13.68 years), seven VBM studies (eight datasets) comprising 262 CS patients (50.32 ± 11.54 years) and 221 HCs (49.13 ± 12.30 years), as well as 1 DTI study with 42 CS patients (42.8 ± 9.3 years) and 42 HCs (42.4 ± 9.4 years). However, no SBM studies were included in this research. In the rs-fMRI cohort, eight studies focused on cervical spondylotic myelopathy (CSM) (Tan et al., 2015; Chen Z. et al., 2018; Kuang and Zha, 2019; Ge et al., 2021; Fan et al., 2022; Zhao et al., 2022; Su et al., 2023; Wu et al., 2024), three on cervical spondylotic radiculopathy (CSR) (Yu et al., 2017; Xu et al., 2018; Yue and Du, 2020), and the remaining two on pure spondylosis (Chen J. et al., 2018; Bai et al., 2022), while the VBM cohort included four studies on CSM (Chen et al., 2022; Tian et al., 2023; Kuang and Zha, 2024; Wang et al., 2024), one study on CSR (Yu et al., 2017), and the remaining two focusing on pure spondylosis (Bernabéu-Sanz et al., 2020; Yang et al., 2020). Besides, among the 13 rs-fMRI studies, one research used fALFF method (Wu et al., 2024), four studies utilized ReHo method (Tan et al., 2015; Yu et al., 2017; Chen J. et al., 2018; Xu et al., 2018), six studies employed ALFF method (Yue and Du, 2020; Ge et al., 2021; Bai et al., 2022; Fan et al., 2022; Zhao et al., 2022; Su et al., 2023), while the remaining two researches utilized both ReHo and ALFF methods (Chen Z. et al., 2018; Kuang and Zha, 2019). In cases where both ReHo and ALFF were used, only results from the more statistically significant method were included in our meta-analysis. The quality assessment of the included studies indicated acceptable levels, with each study scoring at least 17 points (Wang et al., 2018). Details of the included studies were presented in Table 1.

**Table 1:** Research included in this study.

**Research**	**Diagnostic methods**	**Methods**	**CS**	**Subjects (Female)**		**Mean age (SD)**		**Statistical threshold**	**Research design**	**Quality scores**
				**Patients**	**HCs**	**Patients**	**HCs**			
**Research included in rs-fMRI meta-analysis**										
Bai et al. (2022)	CT & MR	ALFF	CS	31 (15)	31 (15)	51.79 (10.21)	51.52 (9.84)	*p* ≤ 0.001, FWE corrected	Longitudinal study	19
Chen J. et al. (2018)	NPQ	ReHo	CS	104 (59)	96 (46)	24.90 (1.98)	24.80 (1.52)	*p* < 0.005, FWE corrected	Longitudinal study	20
Chen Z. et al. (2018)	JOA	ReHo^a^	CSM	27 (12)	11 (5)	57.90 (9.10)	54.80 (8.40)	*P* < 0.05, FWE corrected	Longitudinal study	19
Fan et al. (2022)	JOA	ALFF	CSM	44 (22)	38 (18)	51.30 (2.8)	51.70 (3.6)	*P* < 0.05, FWE corrected	Longitudinal study	19
Ge et al. (2021)	JOA	ALFF	CSM	12 (7)	14 (8)	55.42 (10.58)	50.05 (11.52)	*P* < 0.05, AlphaSim corrected	Cross-sectional study	19
Kuang and Zha (2019)	JOA	ALFF^b^	CSM	33 (17)	33 (18)	54.78 (8.41)	53.52 (8.13)	*P* < 0.01, FDR corrected	Cross-sectional study	20
Su et al. (2023)	JOA	ALFF	CSM	62 (31)	60 (30)	53.3 (7.38)	53.4 (7.47)	*p* ≤ 0.05, FWE corrected	Longitudinal study	18
Tan et al. (2015)	JOA & NDI	ReHo	CSM	21 (8)	21 (8)	47.95 (7.00)	47.90 (7.00)	*P* < 0.05, AlphsSim corrected	Cross-sectional study	19
Wu et al. (2024)	JOA	fALFF	CSM	20 (11)	20 (13)	53.50 (9.27)	49.20 (11.06)	*P* < 0.005, GRF corrected	Cross-sectional study	18
Xu et al. (2018)	MRI	ReHo	CSR	25 (11)	20 (11)	47.70 (11.00)	42.50 (11.90)	*P* < 0.01, AlphaSim corrected	Cross-sectional study	17
Yu et al. (2017)	MRI/CT	ReHo	CSR	25 (12)	20 (10)	47.68 (10.99)	42.50 (11.94)	*p* < 0.01, AlphaSim corrected	Cross-sectional study	19
Yue and Du (2020)	MRI	ALFF	CSR	28 (11)	25 (12)	47.04 (8.74)	43.56 (11.96)	*p* < 0.05, AlphaSim corrected	Cross-sectional study	20
Zhao et al. (2022)	JOA	ALFF	CSM	21 (10)/33(17)	11 (5)/39(19)	53.3 (9.13)/53.5 (11.9)	54.8 (8.4)/53.7 (8.3)	*P* ≤ 0.05, FWE corrected	Cross-sectional study	20
**Research included in VBM meta-analysis**										
Bernabéu-Sanz et al. (2020)	mJOHA	VBM	CS	27 (14)	24 (12)	55.92 (11.98)	55.79 (12.12)	*P* < 0.001, FDR corrected	Cross-sectional study	17
Chen et al. (2022)	mJOA	VBM	CSM	10 (6)/10 (6)	10 (6)	52.1 (3.78)/52.7 (4.62)	52.7 (6.67)	*p* < 0.05, FWE corrected	Cross-sectional study	20
Kuang and Zha (2024)	mJOA	VBM	CSM	40 (25)	28(17)	40.20 (10.12)	39.54 (10.86)	*P* < 0.001, FWE corrected	Cross-sectional study	19
Tian et al. (2023)	MRI & MR & JOA	VBM	CSM	62 (31)	42 (21)	57.2 (8.17)	57.1 (8.25)	*P* < 0.05, FDR corrected	Cross-sectional study	19
Wang et al. (2024)	MRI & JOA	VBM	CSM	57 (23)	57 (26)	52.7 (12.4)	50.9 (13.6)	*P* < 0.05, FDR corrected	Cross-sectional study	19
Yang et al. (2020)	MRI	VBM	CS	31 (11)	30 (10)	41.19 (1.67)	40.27 (1.85)	Uncorrected	Cross-sectional study	18
Yu et al. (2017)	MRI/CT	VBM	CSR	25 (12)	20 (10)	47.68 (10.99)	42.50 (11.94)	*P* < 0.05 NA	Cross-sectional study	19
**DTI research**										
Li et al. (2022)	X-ray, MRI & JOA	DTI-TBSS	CS	42 (28)	42 (28)	42.8 (9.3)	42.4 (9.4)	*P* < 0.05, FWE corrected	Cross-sectional study	19

rs-fMRI, resting-state functional magnetic resonance imaging; CS, cervical spondylosis; HCs, healthy controls; SD, standard deviation; CT, Computed tomography; MR, magnetic resonance scanning; NPQ, neck pain questionnaire; JOA, The Japanese Orthopedic Association scale; NDI, Neck Disability Index Scores; MRI, Magnetic Resonance Imaging; ALFF, amplitude of low-frequency fluctuations; ReHo, regional homogeneity; DCSM, degenerative cervical spondylotic myelopathy; CSR, cervical spondylotic radiculapathy; FWE, family wise error; FDR, false discovery rate; VBM, voxel-based morphometry; mJOHA, Modified Japanese Orthopedic Association Scoring System; mJOA, Modified Japanese Orthopedic Association; NA, not available; GRF, Gaussian random field; TBSS, tract-based spatial statistics.

^a^Both ALFF and ReHo analyses were conducted in this study, and the results of ReHo analysis were included in our meta-analysis.

^b^Both ALFF and ReHo analyses were conducted in this study, and the results of ALFF analysis were included in our meta-analysis.

### 3.2 Functional and structural alterations

**Figure 2:**
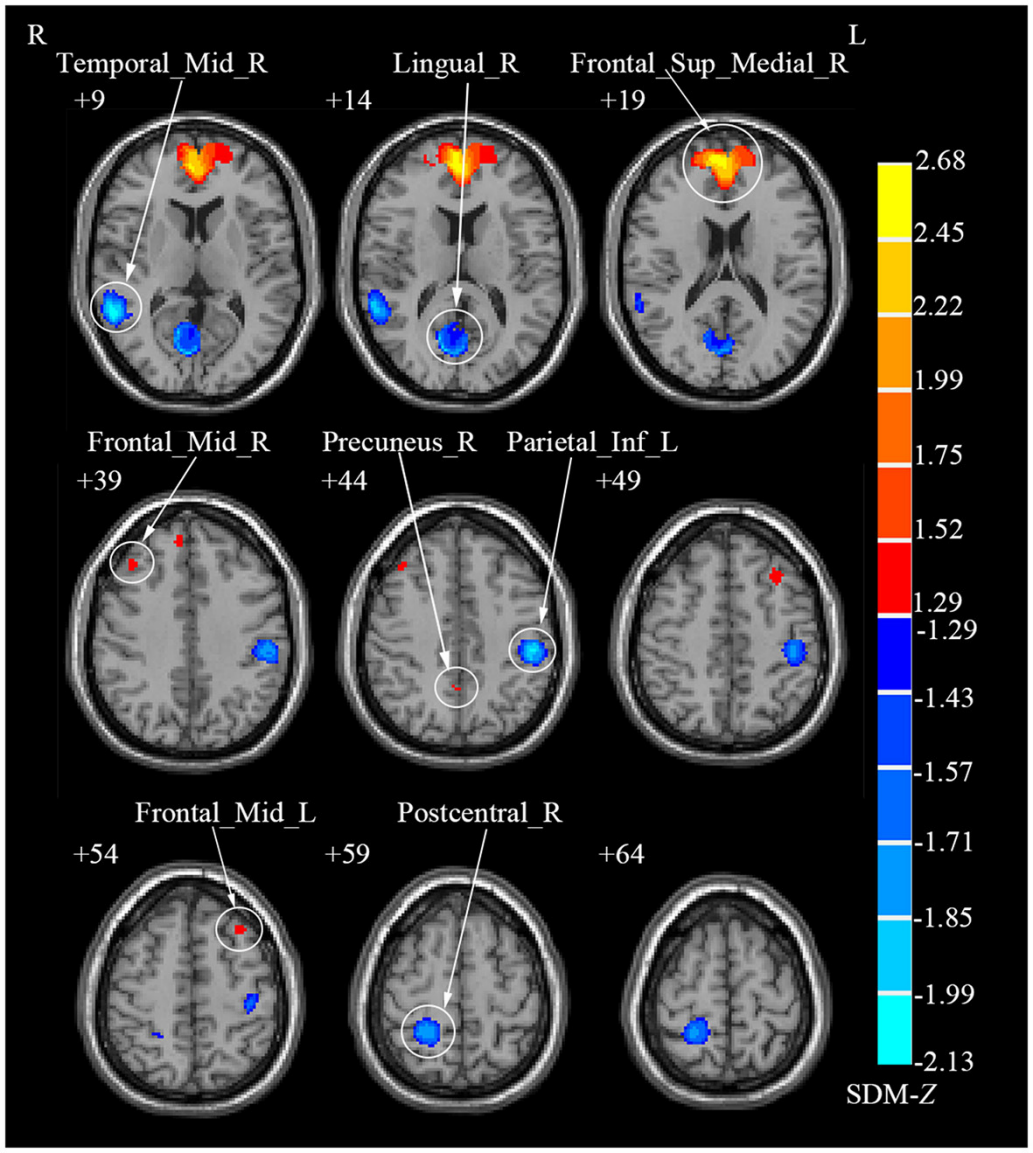
Brain regions showing increased and decreased regional spontaneous brain activities in patients with cervical spondylosis compared to healthy controls. Frontal_Sup_Medial_R, right superior frontal gyrus, medial; Frontal_Mid_L, left middle frontal gyrus; Precuneus_R, right precuneus; Frontal_Mid_R, right middle frontal gyrus; Lingual_R, right lingual gyrus; Temporal_Mid_R, right middle temporal gyrus; Parietal_Inf_L, left inferior parietal gyri; Postcentral_R, right postcentral gyrus.

**Figure 3:**
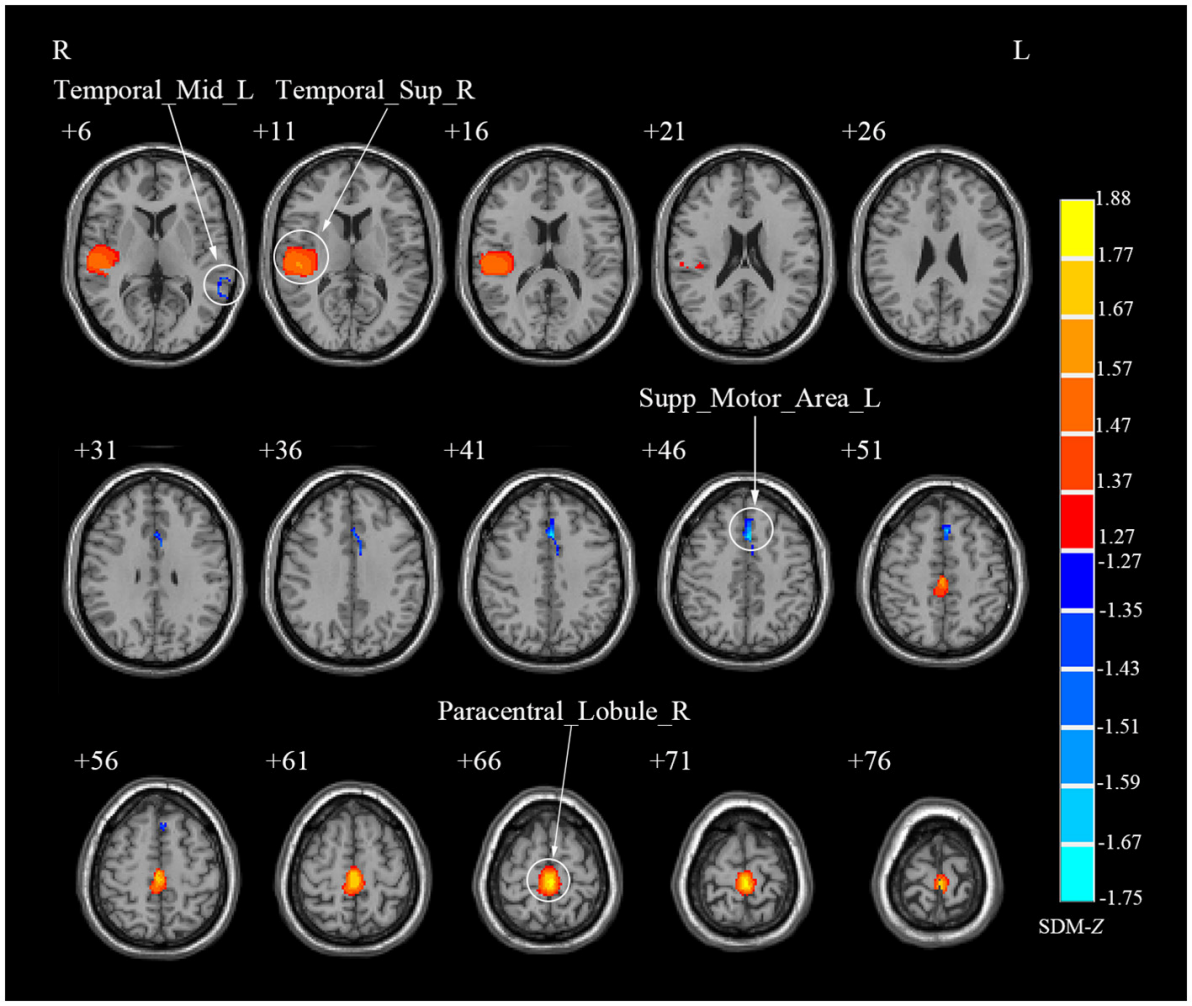
Brain regions showing abnormal gray matter volume in patients with cervical spondylosis compared to healthy controls. Temporal_Sup_R, right superior temporal gyrus; Paracentral_Lobule_R, right paracentral lobule; Supp_Motor_Area_L, left supplementary motor area; Temporal_Mid_L, left middle temporal gyrus.

Due to the small sample size of DTI study (*N* = 1), the meta-analysis was not conducted for this modality. Specifically, meta-analysis was conducted only for rs-fMRI and VBM studies. In rs-fMRI meta-analysis, the results showed that decreased activities were found in the right lingual gyrus (LING), right middle temporal gyrus (MTG), left inferior parietal gyri (IPL) and right postcentral gyrus (PoCG) among CS patients when compared to HCs, while increased activities were detected in the right medial superior frontal gyrus (SFGmed), bilateral MFG and right precuneus (PCUN) among CS patients (Table 2; Figure 2). Complementing these findings, the VBM meta-analysis revealed that compared to HCs, CS patients demonstrated increased GMV in the right superior temporal gyrus (STG) and right paracentral lobule (PCL) while decreased GMV in the left supplementary motor area (SMA) and left MTG (Table 3; Figure 3). These alterations highlight significant functional and structural differences in CS patients.

**Table 2:** Regions with abnormal regional spontaneous brain activities in CS patients relative to HCs.

**Anatomical label**	**Peak MNI coordinate**	**Cluster**		**SDM-Z value**	***P*-value**	**Effect size**	**Jacknife sensitivity analysis**	**Heterogeneity**	**Egger test (*p*-value)**
	**(*****x***, ***y***, ***z*****)**	**Size**	**Breakdown (size)**						
**CS** > **HCs**									
Frontal_Sup_Medial_R (aal)	6, 62, 18	3,035	• Frontal_Sup_Medial_L (992) • Frontal_Sup_Medial_R (917) • Frontal_Sup_L (333) • Frontal_Sup_R (250) • Cingulum_Ant_L (188) • Cingulum_Ant_R (144) • Frontal_Med_Orb_L (107) • Frontal_Med_Orb_R (43) • Frontal_Mid_R (14)	2.685	~0	0.177511	13/13	Yes	0.931
Frontal_Mid_L (aal)	−30, 24, 50	52	Frontal_Mid_L (52)	1.520	0.001131773	0.122292	11/13	No	0.099
Frontal_Mid_R (aal)	42, 34, 42	28	Frontal_Mid_R (28)	1.424	0.002167523	0.145177	11/13	No	0.154
Precuneus_R (aal)	4, −48, 46	13		1.351	0.003453374	0.089147	11/13	No	0.466
**CS**<**HCs**									
Lingual_R (aal)	14, −60, 8	886	• Calcarine_R (401) • Calcarine_L (138) • Lingual_R (119) • Cuneus_R (85) • Precuneus_R (58) • Cuneus_L (40) • Precuneus_L (16) • Lingual_L (13)	−1.932	0.000266016	−0.127676	12/13	Yes	0.322
Temporal_Mid_R (aal)	58, −48, 8	635	• Temporal_Mid_R (373) • Temporal_Sup_R (254)	−2.128	0.000082314	−0.140537	12/13	No	0.066
Parietal_Inf_L (aal)	−48, −26, 44	545	• Postcentral_L (269) • Parietal_Inf_L (222) • SupraMarginal_L (48)	−2.059	0.000122070	−0.135972	12/13	No	0.039^*^
Postcentral_R (aal)	24, −46, 60	360	• Postcentral_R (198) • Parietal_Sup_R (137)	−1.831	0.000463724	−0.190518	12/13	No	0.114

^*^The publication bias was detected in the cluster of Parietal_Inf_L by Egger test.

CS, cervical spondylosis; HCs, healthy controls; MNI, Montreal Neurological Institute; SDM, Signed Differential Mapping; Frontal_Sup_Medial_R, right superior frontal gyrus, medial; Frontal_Mid_L, left middle frontal gyrus; Precuneus_R, right precuneus; Frontal_Mid_R, right middle frontal gyrus; Lingual_R, right lingual gyrus; Temporal_Mid_R, right middle temporal gyrus; Parietal_Inf_L, left inferior parietal gyri; Postcentral_R, right postcentral gyrus; Frontal_Sup_Medial_L, left superior frontal gyrus, medial; Frontal_Sup_L, left superior frontal gyrus, dorsolateral; Frontal_Sup_R, right superior frontal gyrus, dorsolateral; Cingulum_Ant_L, left anterior cingulate and paracingulate gyri; Cingulum_Ant_R, right anterior cingulate and paracingulate gyri; Frontal_Med_Orb_L, left superior frontal gyrus, medial orbital; Frontal_Med_Orb_R, right superior frontal gyrus, medial orbital; Calcarine_R, right calcarine fissure and surrounding cortex; Calcarine_L, left calcarine fissure and surrounding cortex; Cuneus_R, right cuneus; Cuneus_L, left cuneus; Precuneus_L, left precuneus; Lingual_L, left lingual gyrus; Temporal_Sup_R, right superior temporal gyrus; Postcentral_L, left postcentral gyrus; SupraMarginal_L, left supramarginal gyrus; Parietal_Sup_R, right superior parietal gyrus.

Regions with < 10 voxels are not reported in the cluster breakdown.

**Table 3:** Regions with abnormal gray matter volume in patients with CS compared to HCs.

**Anatomical label**	**Peak MNI coordinate**	**Cluster**		**SDM-Z value**	***P*-value**	**Effect size**	**Jacknife sensitivity analysis**	**Heterogeneity**	**Egger test (*p*-value)**
	**(*****x***, ***y***, ***z*****)**	**Size**	**Breakdown (size)**						
**CS** > **HCs**									
Temporal_Sup_R (aal)	50, −24, 10	1,738	• Temporal_Sup_R (757) • Rolandic_Oper_R (472) • Insula_R (234) • Heschl_R (220) • SupraMarginal_R (36)	1.575	0.000326395	0.296620	7/8	No	0.533
Paracentral_Lobule_R (aal)	0, −30, 70	1,328	• Paracentral_Lobule_L (534) • Paracentral_Lobule_R (410) • Supp_Motor_Area_R (161) • Precuneus_L (50) • Supp_Motor_Area_L (35) • Cingulum_Mid_R (31) • Precuneus_R (25) • Cingulum_Mid_L (13)	1.875	0.000028670	0.525251	8/8	Yes	0.075
**CS**<**HCs**									
Supp_Motor_Area_L (aal)	−2, 18, 44	414	• Frontal_Sup_Medial_L (150) • Supp_Motor_Area_L (130) • Cingulum_Mid_L (83) • Cingulum_Ant_L (25) • Frontal_Sup_Medial_R (11)	−1.751	0.000131071	−0.161335	8/8	No	0.228
Temporal_Mid_L (aal)	−58, −46, 6	157	Temporal_Mid_L (146)	−1.452	0.000582159	−0.133682	7/8	No	0.248

CS, cervical spondylosis; HCs, healthy controls; MNI, Montreal Neurological Institute; SDM, Signed Differential Mapping; Temporal_Sup_R, right superior temporal gyrus; Paracentral_Lobule_R, right paracentral lobule; Supp_Motor_Area_L, left supplementary motor area; Temporal_Mid_L, left middle temporal gyrus; Rolandic_Oper_R, right rolandic operculum; Insula_R, right insula; Heschl_R, right heschl gyrus; SupraMarginal_R, right supramarginal gyrus; Paracentral_Lobule_L, left paracentral lobule; Supp_Motor_Area_R, right supplementary motor area; Precuneus_L, left precuneus; Cingulum_Mid_R, right median cingulate and paracingulate gyri; Precuneus_R, right precuneus; Cingulum_Mid_L, left median cingulate and paracingulate gyri; Frontal_Sup_Medial_L, left superior frontal gyrus, medial; Cingulum_Ant_L, left anterior cingulate and paracingulate gyri; Frontal_Sup_Medial_R, right superior frontal gyrus, medial.

Regions with < 10 voxels are not reported in the cluster breakdown.

### 3.3 Multi-modal meta-analysis

**Figure 4:**
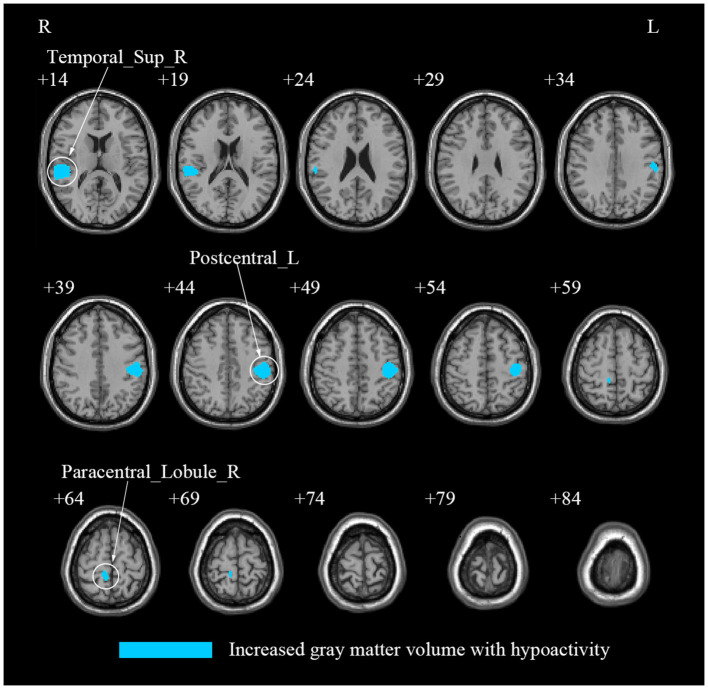
A multi-modal meta-analysis of combined structural and functional alterations in patients with cervical spondylosis. Postcentral_L, left postcentral gyrus; Temporal_Sup_R, right superior temporal gyrus; Paracentral_Lobule_R, right paracentral lobule; Parietal_Inf_L, left inferior parietal, but supramarginal and angular gyri; Precentral_L, left precentral gyrus; SupraMarginal_L, left supramarginal gyrus; Rolandic_Oper_R, right rolandic operculum; SupraMarginal_R, right supramarginal gyrus; Temporal_Mid_R: right middle temporal gyrus; Heschl_R, right heschl gyrus; Postcentral_R, right postcentral gyrus; Precentral_R, right precentral gyrus; Precuneus_R, right precuneus.

Our multi-modal meta-analysis revealed a unique pattern in CS patients when compared to HCs. Specifically, compared to HCs, CS patients showed a conjoint increase of GMV and decreased regional spontaneous brain activity in the left PoCG, right STG and right PCL (Table 4; Figure 4; Supplementary Table S5; Supplementary Figures S2, S3).

**Table 4:** Multi-modal structural and functional alterations in CS patients compared to HCs.

**Description**	**Peak MNI coordinate**	**Cluster size**	**Cluster breakdown (no. of voxels)**
	**(*****x***, ***y***, ***z*****)**		
**Increased GMV** + **hypoactivity**			
Postcentral_L (aal)	−48, −20, 44	1,046	• Postcentral_L (678) • Parietal_Inf_L (194) • Precentral_L (101) • SupraMarginal_L (73)
Temporal_Sup_R (aal)	60, −34, 14	948	• Temporal_Sup_R (634) • Rolandic_Oper_R (181) • SupraMarginal_R (76) • Temporal_Mid_R (35) • Heschl_R (19)
Paracentral_Lobule_R (aal)	12, −40, 64	110	• Paracentral_Lobule_R (74) • Postcentral_R (31)

CS, cervical spondylosis; HCs, healthy controls; MNI, Montreal Neurological Institute; GMV, gray matter volume; Postcentral_L, left postcentral gyrus; Temporal_Sup_R, right superior temporal gyrus; Paracentral_Lobule_R, right paracentral lobule; Parietal_Inf_L, left inferior parietal, but supramarginal and angular gyri; Precentral_L, left precentral gyrus; SupraMarginal_L, left supramarginal gyrus; Rolandic_Oper_R, right rolandic operculum; SupraMarginal_R, right supramarginal gyrus; Temporal_Mid_R, right middle temporal gyrus; Heschl_R, right heschl gyrus; Postcentral_R, right postcentral gyrus; Precentral_R, right precentral gyrus; Precuneus_R, right precuneus.

Regions with < 10 voxels are not reported in the cluster breakdown.

### 3.4 Analyses of jackknife sensitivity, heterogeneity, and publication bias

The jackknife sensitivity analysis affirmed the reliability of our findings. In rs-fMRI meta-analysis, the right SFGmed emerged as the most consistently altered region, showing replicable changes across all 13 dataset combinations (Table 2). The right LING, right MTG, left IPL and right PoCG demonstrated significant alterations in 12/13 combinations. Besides, the bilateral MFG and right PCUN also showed significant changes in 11/13 combinations. In VBM meta-analysis, the right PCL and left SMA displayed altered GMV consistently across all study combinations while the right STG and left MTG exhibited altered GMV in 7/8 dataset combinations (Table 3).

In rs-fMRI meta-analysis, we identified significant heterogeneity in the right SFGmed and right LING (Table 2). Similarly, in the VBM meta-analysis, the right PCL displayed significant heterogeneity (Table 3). Furthermore, our Egger test only revealed publication bias in the left IPL through rs-fMRI meta-analysis (*p* = 0.039) (Table 2).

### 3.5 Subgroup analyses

**Figure 5:**
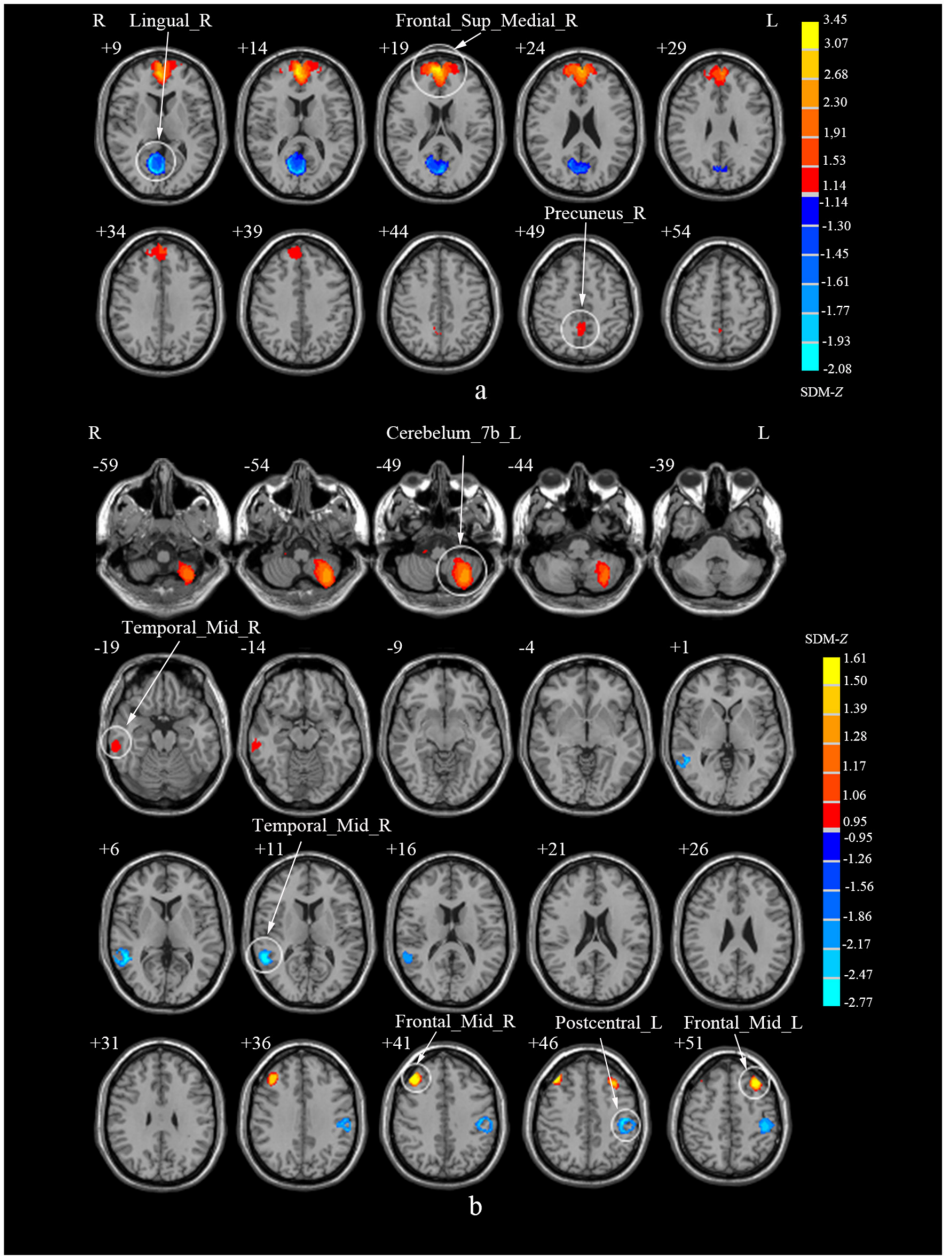
Subgroup analyses of ALFF and ReHo studies in CS patients compared to HCs. **(a)** Subgroup analysis of ALFF studies. **(b)** Subgroup analysis of ReHo studies. ALFF, amplitude of low-frequency fluctuations; ReHo, regional homogeneity; CS, cervical spondylosis; HCs, healthy controls; Frontal_Sup_Medial_R, right superior frontal gyrus, medial; Precuneus_R, right precuneus; Lingual_R, right lingual gyrus; ReHo, regional homogeneity; Cerebellum_7b_L, left cerebellum, hemispheric lobule VIIB; Frontal_Mid_R, right middle frontal gyrus; Frontal_Mid_L, left middle frontal gyrus; Temporal_Mid_R, right middle temporal gyrus; Postcentral_L, left postcentral gyrus; Temporal_Mid_R, right middle temporal gyrus.

**Figure 6:**
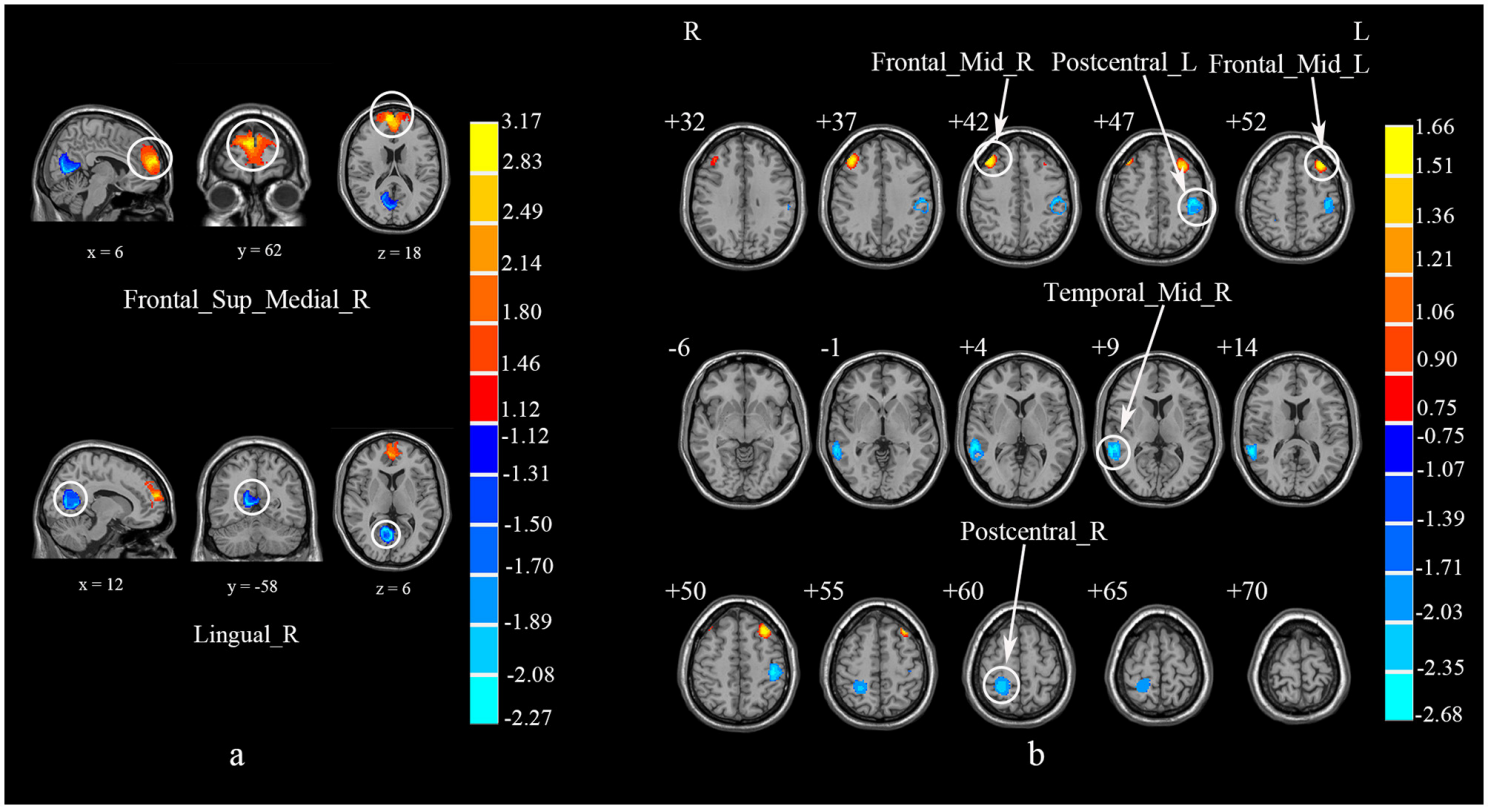
Subgroup analyses of CSM studies and studies on CS patients without myelopathy. **(a)** Subgroup meta-analysis of CSM studies. **(b)** Subgroup meta-analysis of studies on CS patients without myelopathy. CSM, cervical spondylotic myelopathy; CS, cervical spondylosis; Frontal_Sup_Medial_R, right superior frontal gyrus, medial; Calcarine_R, right Calcarine fissure and surrounding cortex; Frontal_Mid_R, right middle frontal gyrus; Frontal_Mid_L, left middle frontal gyrus; Temporal_Mid_R, right middle temporal gyrus; Postcentral_L, left postcentral gyrus; Postcentral_R, right postcentral gyrus.

The subgroup analysis of ALFF (*N* = 7) revealed that compared to HCs, CS patients showed increased activities in the right SFGmed and PCUN, but decreased activity in the right LING (Table 5; Figure 5a). The subgroup analysis of ReHo (*N* = 5) indicated increased activities in the left cerebellum lobule VIIB, bilateral MFG and right MTG, while decreased activities in the left PoCG and right MTG in CS (Table 5; Figure 5b). Subgroup analysis on CSM studies (*N* = 8) showed that compared to HCs, CSM patients demonstrated decreased brain activity in the right LING while increased brain activity in the right SFGmed (Table 6; Figure 6a). Additionally, the subgroup analysis of studies measuring differences between HCs and CS patients without myelopathy (*N* = 5) detected decreased brain activities in the right MTG and bilateral PoCG, while increased neural activities in the bilateral MFG among CS patients when compared to HCs (Table 6; Figure 6b). The findings revealed different impacts of the analytical methods and conditions of the disease.

**Table 5:** Subgroup meta-analysis of ALFF and ReHo studies in CS patients compared to HCs.

**Anatomical label**	**Peak MNI coordinate**	**Cluster size**	**SDM-Z value**	***P-*value**	**Effect size**
	**(*****x***, ***y***, ***z*****)**				
**Subgroup analysis of ALFF studies**					
**CS** > **HCs**					
Frontal_Sup_Medial_R (aal)	6, 62, 18	3,645	3.454	~0	0.315417
Precuneus_R (aal)	4, −54, 48	143	1.624	0.000910342	0.142532
**CS**<**HCs**					
Lingual_R (aal)	6, −62, 4	1,676	−2.082	0.000013649	0.182943
**Subgroup meta-analysis of ReHo studies**					
**CS** > **HCs**					
Cerebellum_7b_L (aal)	−36, −66, −50	1,517	1.394	0.000158429	0.245411
Frontal_Mid_R (aal)	42, 34, 42	298	1.611	0.000014722	0.465401
Frontal_Mid_L (aal)	−32, 28, 54	231	1.575	0.000027359	0.384599
Temporal_Mid_R (aal)	66, −30, −16	160	1.074	0.001376927	0.270274
**CS**<**HCs**					
Postcentral_L (aal)	−44, −26, 48	597	−2.613	0.000134945	−0.276781
Temporal_Mid_R (aal)	54, −50, 10	481	−2.771	0.000059605	−0.293831

ALFF, amplitude of low-frequency fluctuation; CS, cervical spondylosis; HCs, healthy controls; MNI, Montreal Neurological Institute; SDM, Signed Differential Mapping; Frontal_Sup_Medial_R, right superior frontal gyrus, medial; Precuneus_R, right precuneus; Lingual_R, right lingual gyrus; ReHo, regional homogeneity; Cerebellum_7b_L, left cerebellum, hemispheric lobule VIIB; Frontal_Mid_R, right middle frontal gyrus; Frontal_Mid_L, left middle frontal gyrus; Temporal_Mid_R, right middle temporal gyrus; Postcentral_L, left postcentral gyrus; Temporal_Mid_R, right middle temporal gyrus.

**Table 6:** Subgroup meta-analysis of CSM patients and CS patients with/without radiculopathy, respectively.

**Anatomical label**	**Peak MNI coordinate**	**Cluster size**	**SDM-Z value**	***P*-value**	**Effect size**
	**(*****x***, ***y***, ***z*****)**				
**Subgroup meta-analysis of CSM patients**					
**CS** > **HCs**					
Frontal_Sup_Medial_R (aal)	6, 62, 18	2,643	3.169	0.000000536	0.177511
**CS**<**HCs**					
Lingual_R (aal)	12, −58, 6	1,378	−2.273	0.000000775	−0.112792
**Subgroup meta-analysis of pure spondylosis and cervical radiculopathy**					
**CS** > **HCs**					
Frontal_Mid_R (aal)	42, 34, 42	351	1.664	~0	0.442569
Frontal_Mid_L (aal)	−32, 28, 54	302	1.600	~0	0.360306
**CS**<**HCs**					
Temporal_Mid_R (aal)	62, −40, 4	787	−2.675	0.000060618	−0.268904
Postcentral_L (aal)	−48, −22, 48	632	−2.532	0.000110686	−0.253810
Postcentral_R (aal)	26, −46, 60	580	−2.146	0.000720203	−0.539957

CSM, cervical spondylotic myelopathy; CS, cervical spondylosis; MNI, Montreal Neurological Institute; SDM, Signed Differential Mapping; HCs, healthy controls; Frontal_Sup_Medial_R, right superior frontal gyrus, medial; Calcarine_R, right Calcarine fissure and surrounding cortex; Frontal_Mid_R, right middle frontal gyrus; Frontal_Mid_L, left middle frontal gyrus; Temporal_Mid_R, right middle temporal gyrus; Postcentral_L, left postcentral gyrus; Postcentral_R, right postcentral gyrus.

### 3.6 Meta-regression analyses

The meta-regression analyses revealed that increased mean age of patients in rs-fMRI studies (available in all rs-fMRI studies) was associated with decreased regional spontaneous brain activity in the left IPL (MNI coordinates: −52, −28, 42; Number of voxels: 55; Peak intensity: 3.658; *p* = 0.000053644), but the percentage of female (available in all rs-fMRI studies) and the JOA score of patients (available in seven rs-fMRI studies) were not associated with CS-related regional spontaneous brain activity changes. Besides, the percentage of female patients (available in all VBM studies), the mean age (available in all VBM studies) and the JOA scores (available in five VBM studies) of patients were not associated with CS-related GMV alterations.

## 4 Discussion

This study represented the first comprehensive meta-analysis that investigated the consistent alterations in brain structure and function among CS patients by combining voxel-based rs-fMRI and VBM studies. Although the meta-analyses of SBM and DTI studies were not conducted due to the insufficient sample sizes, previous studies still indicated that these measures also provided alternative neuroimaging approach for assessing the pathophysiological features of CS. The main discoveries of the present study are as follows: (1) The rs-fMRI meta-analysis indicated significantly lower regional spontaneous brain activities in the right LING, right MTG, left IPL and right PoCG, while higher brain activities in the right SFGmed, bilateral MFG and right PCUN among CS patients when compared to HCs. (2) The VBM meta-analysis revealed decreased GMV in the left SMA and MTG while increased GMV within the right STG and PCL in CS patients. (3) Our multi-modal meta-analysis uncovered conjoint increased GMV but decreased regional brain activity in the left PoCG, right STG and right PCL.

In CS patients, decreased regional spontaneous brain activity was noted in the right LING, right MTG, left IPL and right PoCG, while increased regional spontaneous brain activity was observed in the right PCUN, right SFGmed, and bilateral MFG. The right LING, belonging to the primary visual cortex (V1), is responsible for receiving and transmitting visual stimulation (Chang et al., 2023). The hypoactivity of the right LING may suggest the visual processing impairment in CS patients since studies have found the symptom of blurred vision in CS patients, especially in those with CSM (Sun et al., 2013; Sun Y. et al., 2016; Chen Z. et al., 2018), which could also explain our finding of hypoactivity in the right LING by the subgroup analysis of studies on CSM patients. In other words, we speculate that the activation of this brain region in different groups may be related to the severity of the disease. Considering that CSM is not a primary visual disorder, the right LING may be a potential neurobiological marker for the diagnosis and recovery of CSM (Takenaka et al., 2019). In addition, our subgroup meta-analysis of methods revealed that this brain area also depended on the analytical method of ALFF. This may indicate strong regional spontaneous activity in this brain area at the single-voxel level, rather than synchronized activity with neighboring voxels, since ALFF gauges the intensity of spontaneous brain activity within a single voxel (Zang et al., 2007; Jia et al., 2020), while ReHo measures the similarity of time courses within clusters consisting of 27 adjacent voxels (Song et al., 2011).

The MTG, IPL, and PCUN are key constituents of the default mode network which involves cognitive and emotional processing (Usui et al., 2020; Yue and Du, 2020; Wu et al., 2023). Specifically, the MTG takes part in diverse functions including cognitive, visual and sensory processing due to its anterior association with the visual network and default mode network (Wu et al., 2020). Studies have indicated that CS patients would suffer from memory loss, poor attention, depression and the absence of visual cues (Theodore, 2020; Zhao et al., 2020). The findings of decreased regional spontaneous brain activity and GMV in the right MTG may be associated with the above symptoms or disorders of CS patients, explaining their underlying neurophysiological bases, since previous studies indicated that these symptoms could not be explained by the degeneration of cervical spine alone (Sun et al., 2018; Fard et al., 2024). The IPL, a key part of the parietal-integrated region and somatosensory association cortex, is involved in translating various sensory modes into actions, namely, sensorimotor transformation (Zhou et al., 2015; Chang et al., 2023). For example, Patri et al. (2020) noted that IPL disruption can impair its role in actualizing motor intentions. Scholars also reported loss of manual dexterity as well as impaired gait and balance in CS patients (Theodore, 2020), so the hypoactivity of the left IPL may indicate patient's impaired function in sensorimotor transformation. Furthermore, our meta-regression analysis revealed that older patients were significantly more likely to report decreased regional spontaneous brain activity in this area, which may provide evidence for the diagnosis of CS in elderly populations. This was consistent with previous studies which also detected the association between age and the prognosis of CS (Lv Y. et al., 2018). However, the publication bias was detected in this area, which may be caused by incomplete research data (Cheng et al., 2023) or underlying differences between smaller and larger studies (Egger et al., 1997; Lau et al., 2006), so the results should be interpreted with scrutiny (Wang et al., 2022). Further studies are needed in the future to verify the functional brain alterations in the left IPL. The PCUN is linked to advanced cognitive functions like episodic memory and visuospatial imagination (Cavanna and Trimble, 2006). Woodworth et al. (2019) also found decreased cortical thickness in the bilateral PCUN among patients with cervical myelopathy. In view of the cognitive impairments reported in CS patients, like hypomnesia and affective disturbance, the hyperactivity of the right PCUN may be a functional compensation for its structural impairment in dealing with cognitive information.

The SFGmed and MFG, integral to the prefrontal lobe, are responsible for diverse functions encompassing sensation, emotion and cognition (Garcia-Larrea and Peyron, 2013). Prior research indicated that the prefrontal cortex integrates sensory and emotional pain information through modulating cortex-subcortical and intercortical nociceptive pathways (Yang et al., 2013). It was also found that the region was involved in processing cognition and negative emotions (Tyborowska et al., 2018; Magon et al., 2019). For example, Gong et al. (2020) found that the heightened ALFF in this area was related to major depression and bipolar disorder. While neck pain is the predominant symptom in CS patients (Takagi et al., 2011), this discomfort, along with other CS-associated ailments, could elicit negative emotions, typically represented by anxiety and depression (Zhao et al., 2020; Chu et al., 2022; Pei et al., 2022). Furthermore, scholars also found cortical thinning in the sensorimotor and pain-related areas (e.g., the superior frontal cortex) of patients (Wang et al., 2024). Therefore, the increased brain activity in the SFGmed and MFG may be an indication of their compensatory role in regulating neck pain, negative emotions and cognitive disorders in CS patients. The results also supported the findings of previous study that the regional brain activity in the superior frontal cortex had impact on the prognosis of patients (Fan et al., 2022). It is worth noting that the right SFGmed also demonstrated significant heterogeneity in the rs-fMRI meta-analysis. Our exploratory subgroup analyses revealed that this region was also dependent on the method of ALFF and the stage of cervical myelopathy, similar to the heterogeneity sources of right LING. As stated in previous studies, patients with pure spondylosis usually have symptoms of neck pain while patients with cervical radiculopathy often reported paresthesia such as tingling, burning or shooting pain (Takagi et al., 2011; Theodore, 2020). However, the sensory impairment in patients with cervical myelopathy is more serious than the first two conditions since CSM patients usually suffer from the symptom of numbness or loss of position sensation (Shedid and Benzel, 2007), namely, they could not feel light touch, pain, temperature, or vibrations, or even not know where their body parts are, which would then weaken their abilities in balance and coordination. In this sense, there may be significant differences in functional brain activity between patients with or without myelopathy in the right SFGmed. Besides, the heterogeneity of this brain region is also derived from the analytical methods of ALFF and ReHo, which may be due to their focus on different characteristics of brain activity (An et al., 2013; Lv H. et al., 2018).

Decreased GMV alone was also found in the left SMA in CS patients. The SMA plays a crucial role in bridging cognitive processes with motor actions (Yu et al., 2017) and managing self-initiated movements (Martín-Signes et al., 2019) by projecting its neuron to the spinal cord (Roy et al., 2009). Specifically, it is primarily involved in self-generated and controlled movement, such as in preparing and executing the practical action sequence (Bhagavatula et al., 2016). Meanwhile, this region is also strongly influenced by other aspects of the movements including attention and performance (Bhagavatula et al., 2016). In view of such symptoms as loss of hand movement flexibility and visual impairment in CS patients (Takagi et al., 2011), decreased GMV in the left SMA among CS patients may represent the underlying neurobiological basis of their motor dysfunction. The PCL, located between the marginal branch of the cingulate sulcus and the paracentral sulcus, controls motor and sensory nerve innervation and is mainly associated with motor of lower extremity, especially with the movement and sensation of the opposite leg and foot (Zhou et al., 2018; Choi et al., 2024). The hypoactivity in this brain area among CS patients, CSM patients in particular, may indicate their decreased sensory and motor abilities, especially in the lower extremity. In addition, scholars also revealed cortical atrophy in the right PCL (Wang et al., 2021), so the increase of GMV in this region may be a compensation for its functional impairment and cortical thinning. Significantly, the additional analysis also revealed heterogeneity in the right PCL. To explore the sources of the heterogeneity, we conducted meta-regression analyses rather than subgroup analyses due to the relatively small sample sizes of the subgroups in VBM studies. No correlation was detected between the three regressor variables, namely, the mean age, the percentage of female and the JOA score and the changes of this region, so the findings of heterogeneity in this area warrant cautious interpretation (Ronaldson et al., 2020).

In addition, we also found an increase in GMV paired with decreased regional spontaneous brain activity in the left PoCG and right STG through the multimodal meta-analysis. The PoCG, representative of the primary somatosensory cortex (S1) (Oni-Orisan et al., 2016), processes and interprets a variety of sensory information from other regions, such as proprioception (Cai et al., 2022) and pain perception (Tseng et al., 2013). The decreased regional spontaneous brain activity in the bilateral PoCG may be a neural signal of sensory disturbance in CS patients, since typical symptoms such as numbness or sensory loss in the feet or hands have been reported in CS patients (Theodore, 2020). Furthermore, previous studies also found significant cortical atrophy and decreased sulcus depth in the PoCG among CS patients (Woodworth et al., 2019; Wang et al., 2021, 2023; Chang et al., 2023). Based on this, the increased GMV of the right PoCG may play a compensatory role in sensory processing. In addition, increased ALFF values in the right PoCG was detected to be related with decreased fractional anisotropy (FA) values at the C2 level of spinal cord, demonstrating the pathophysiological interaction between cerebral cortex and spinal cord (Zhou et al., 2014). These findings could further explain the remodeling of cerebral cortex in response to spinal cord injury and facilitate the clinical treatment decisions of physicians. The STG was also found active in pain-inducing studies, indicating its role in sensory processing (Rottmann et al., 2010). Meanwhile, the cortical thickness of STG among CS patients was also detected to be thinner when compared with HCs (Wang et al., 2023). We speculated that the decreased regional spontaneous brain activity of the right STG in CS patients may contribute to their difficulties in accurately recognizing, integrating and processing sensory information, potentially leading to numbness, paresthesia or sensory loss commonly reported (Theodore, 2020), whereas the increased GMV in STG may represent compensations for its decreased regional spontaneous brain activity and cortical atrophy.

The findings of the present meta-analytic study suggest consistent and core alterations in brain structure and function among CS patients as well as a complex interplay between different neuroimaging modalities, regional spontaneous brain activity and GMV in particular, which could provide evidence and reference for clinical practice. Firstly, neuroimaging technique has been increasingly used to explore brain abnormalities in structure and function among CS patients, which generated diverse and inconsistent results. The consistent brain changes in CS patients identified by this meta-analysis could consolidate new insights that are clinically useful, thus contributing to the understanding of the complex pathophysiology of CS (Tahmasian et al., 2019), especially the underlying neuropathological mechanisms of cervical spondylosis. Secondly, the integration of previous studies may provide conclusive evidence for the diagnosis of CS and improve the diagnostic accuracy, thus reducing the waste of medical resources (Fan et al., 2022). For example, according to Fan et al. (2022), the accuracy of support vector machine analysis and linear analysis which were based on DTI and fMRI data for identifying CS patients from HCs has exceeded 97%. Thirdly, the persistent neuroimaging findings of the present study may provide prognostic insights to physicians and facilitate their treatment decision, thus reducing unnecessary sufferings of patients with CS (Fan et al., 2022; Fard et al., 2024). However, some limitations must be acknowledged. First, due to the cross-sectional nature of the included studies, a causal link between CS and brain abnormalities couldn't be established (Gong et al., 2020). Second, this meta-analysis relies on peak coordinates and effect sizes extracted from published studies rather than on raw statistical brain maps, which could affect the precision of the spatial localization of the reported effects (Radua et al., 2012b; Jiang et al., 2017). Third, our meta-analysis may include some studies without correction for multiple comparisons. However, according to Radua and Mataix-Cols (2009), the inclusion of analyses that were not corrected for multiple comparisons did not bias the likelihood of finding significant results. Future research could include more homogeneous studies and provide a more granular understanding of the mechanisms underlying CS.

## 5 Conclusions

The present study conducted a multi-modal meta-analysis to identify the consistent structural and functional brain alterations in CS patients. The results showed that compared to HCs, CS patients demonstrated a significant change in GMV and regional spontaneous brain activity mainly in the visual cortex, the default mode network and the sensorimotor area, with a complex interplay between the two modalities. These findings could provide fresh insights into the pathophysiology of CS, potentially directing future research on its diagnosis and therapeutic approaches.

## Data availability statement

The original contributions presented in the study are included in the article/Supplementary material, further inquiries can be directed to the corresponding authors.

## Author contributions

LCheng: Conceptualization, Funding acquisition, Supervision, Writing – original draft, Writing – review & editing. JZ: Data curation, Formal analysis, Methodology, Writing – original draft, Writing – review & editing. HX: Data curation, Writing – review & editing. ML: Writing – review & editing. SH: Writing – review & editing. WY: Data curation, Supervision, Validation, Resources, Writing – review & editing. PW: Writing – review & editing. LChen: Writing – review & editing. LZ: Writing – review & editing. XJ: Conceptualization, Funding acquisition, Software, Supervision, Writing – review & editing.
