# Supplementary material for: Abnormalities of brain structure and function in cervical spondylosis: a multi-modal voxel-based meta-analysis
Source: Front Neurosci. 2024 Jun 14;18:1415411. doi: 10.3389/fnins.2024.1415411 (PMC11211609; doi:10.3389/fnins.2024.1415411)
Supplement: Supplementary file 1 [file Data_Sheet_1.docx]

Supplementary Materials

# 1. Supplementary Data

Studies included in rs-fMRI and VBM meta-analysis

| **Study** | | | **Diagnostic** | | **Methods** | | **CS** | | **Subjects (Female)** | | | | | **Mean age (SD)** | | | | | | | **Statistical threshold** | | **Medication status** | | **Quality scores** | | |  |  |
| --- | --- | --- | --- | --- | --- | --- | --- | --- | --- | --- | --- | --- | --- | --- | --- | --- | --- | --- | --- | --- | --- | --- | --- | --- | --- | --- | --- | --- | --- |
|  |  |  |  |  |  |  |  |  | **Patients** | | **HCs** | | | **Patients** | | | **HCs** | | | |  |  |  |  |  |  |  |  |  |
| **Studies included in rs-fMRI meta-analysis** | | | | | | | | | | | | | | | | | | | | | | | | | | | |  |  |
| Bai et al. (2022) | | | CT & MR | | ALFF | | CS | | 31 (15) | | 31 (15) | | | 51.79 (10.21) | | | 51.52 (9.84) | | | | p ≤ 0.001, FWE corrected | | Longitudinal study | | 19 | | |  |  |
| Chen J. et al. (2018) | | | NPQ | | ReHo | | CS | | 104 (59) | | 96 (46) | | | 24.90 (1.98) | | | 24.80 (1.52) | | | | p < 0.005, FWE corrected | | Longitudinal study | | 20 | | |  |  |
| Chen Z. et al. (2018) | | | JOA | | ReHo^a^ | | CSM | | 27 (12) | | 11 (5) | | | 57.90 (9.10) | | | 54.80 (8.40) | | | | P < 0.05, FWE corrected | | Longitudinal study | | 19 | | |  |  |
| Fan et al. (2022) | | | JOA | | ALFF | | CSM | | 44 (22) | | 38 (18) | | | 51.30 (2.8) | | | 51.70 (3.6) | | | | P < 0.05, FWE corrected | | Longitudinal study | | 19 | | |  |  |
| Ge et al. (2021) | | | JOA | | ALFF | | CSM | | 12 (7) | | 14 (8) | | | 55.42 (10.58) | | | 50.05 (11.52) | | | | P < 0.05, AlphaSim corrected | | Cross-sectional study | | 19 | | |  |  |
| Kuang & Zha (2019) | | | JOA | | ALFF^b^ | | CSM | | 33 (17) | | 33 (18) | | | 54.78 (8.41) | | | 53.52 (8.13) | | | | P < 0.01, FDR corrected | | Cross-sectional study | | 20 | | |  |  |
| Su et al. (2023) | | | JOA | | ALFF | | CSM | | 62 (31) | | 60 (30) | | | 53.3 (7.38) | | | 53.4 (7.47) | | | | p ≤ 0.05, FWE corrected | | Longitudinal study | | 18 | | |  |  |
| Tan et al. (2015) | | | JOA & NDI | | ReHo | | CSM | | 21 (8) | | 21 (8) | | | 47.95 (7.00) | | | 47.90 (7.00) | | | | P < 0.05, AlphsSim corrected | | Cross-sectional study | | 19 | | |  |  |
| Wu et al. (2024) | | | JOA | | fALFF | | CSM | | 20 (11) | | 20 (13) | | | 53.50 (9.27) | | | 49.20 (11.06) | | | | P < 0.005, GRF corrected | | Cross-sectional study | | 18 | | |  |  |
| Xu et al. (2018) | | | MRI | | ReHo | | CSR | | 25 (11) | | 20 (11) | | | 47.70 (11.00) | | | 42.50 (11.90) | | | | P < 0.01, AlphaSim corrected | | Cross-sectional study | | 17 | | |  |  |
| Yu et al. (2017) | | | MRI/CT | | ReHo | | CSR | | 25 (12) | | 20 (10) | | | 47.68 (10.99) | | | 42.50 (11.94) | | | | p < 0.01, AlphaSim corrected | | Cross-sectional study | | 19 | | |  |  |
| Yue and Du (2020) | | | MRI | | ALFF | | CSR | | 28 (11) | | 25 (12) | | | 47.04 (8.74) | | | 43.56 (11.96) | | | | p < 0.05, AlphaSim corrected | | Cross-sectional study | | 20 | | |  |  |
| Zhao et al. (2022) | | | JOA | | ALFF | | CSM | | 21 (10)/  33(17) | | 11 (5)/  39(19) | | | 53.3 (9.13)/  53.5 (11.9) | | | 54.8 (8.4)/  53.7 (8.3) | | | | P ≤ 0.05, FWE corrected | | Cross-sectional study | | 20 | | |  |  |
| **Studies included in VBM meta-analysis** | | | | | | | | | | | | | | | | | | | | | | | | | | | |  |  |
| Bernabéu-Sanz et al. (2020) | | mJOHA | | | VBM | | CS | | 27 (14) | | | 24 (12) | | | 55.92 (11.98) | | | 55.79(12.12) | | P < 0.001, FDR corrected | | | Cross-sectional study | | | 17 | | | |
| Chen et al. (2022) | | mJOA | | | VBM | | CSM | | 10 (6)/  10 (6) | | | 10 (6) | | | 52.1 (3.78)/  52.7 (4.62) | | | 52.7 (6.67) | | p < 0.05, FWE corrected | | | Cross-sectional study | | | 20 | | | |
| Kuang & Zha (2024) | | mJOA | | | VBM | | CSM | | 40 (25) | | | 28(17) | | | 40.20 (10.12) | | | 39.54(10.86) | | P < 0.001, FWE corrected | | | Cross-sectional study | | | 19 | | | |
| Tian et al. (2023) | | MRI & MR & JOA | | | VBM | | CSM | | 62 (31) | | | 42 (21) | | | 57.2 (8.17) | | | 57.1 (8.25) | | P < 0.05, FDR corrected | | | Cross-sectional study | | | 19 | | | |
| Wang et al. (2024) | | MRI & JOA | | | VBM | | CSM | | 57 (23) | | | 57 (26) | | | 52.7 (12.4) | | | 50.9 (13.6) | | P < 0.05, FDR corrected | | | Cross-sectional study | | | 19 | | | |
| Yang et al. (2020) | | MRI | | | VBM | | CS | | 31 (11) | | | 30 (10) | | | 41.19 (1.67) | | | 40.27 (1.85) | | uncorrected | | | Cross-sectional study | | | 18 | | | |
| Yu et al. (2017) | | MRI/CT | | | VBM | | CSR | | 25 (12) | | | 20 (10) | | | 47.68 (10.99) | | | 42.50(11.94) | | P < 0.05 NA | | | Cross-sectional study | | | 19 | | | |
| **DTI research** | | |  | |  | |  | |  | | |  | | |  | | |  | | |  | |  | | |  | |  |  |
| Li et al. (2021) | | | X-ray, MRI & JOA | | DTI-TBSS | | CS | | 42 (28) | | | 42 (28) | | | 42.8 (9.3) | | | 42.4 (9.4) | | | P < 0.05, FWE corrected | | Cross-sectional study | | | 19 | |  |  |

**Abbreviations:** rs-fMRI, resting-state functional magnetic resonance imaging; CS, Cervical spondylosis; HCs, Healthy controls; SD, Standard deviation; CT, Computed Tomography; MR, Magnetic resonance scanning; NPQ, Neck pain questionnaire; JOA, The Japanese Orthopedic Association scale; NDI, Neck Disability Index Scores; MRI, Magnetic Resonance Imaging; ALFF, Amplitude of low-frequency fluctuations; ReHo, Regional homogeneity; DCSM, degenerative cervical spondylotic myelopathy; CSR, Cervical spondylotic radiculapathy; FWE, Family wise error; FDR, False discovery rate; VBM, Voxel-based morphometry; mJOHA, Modified Japanese Orthopaedic Association Scoring System; mJOA, Modified Japanese Orthopaedic Association; NA, Not available; GRF, Gaussian random field, TBSS, tract-based spatial statistics.

a Both ALFF and ReHo analyses were conducted in this study, and the results of ReHo analysis were included in our meta-analysis.

b Both ALFF and ReHo analyses were conducted in this study, and the results of ALFF analysis were included in our meta-analysis.

# 2. Supplementary Tables

**Table S1** PRISMA 2020 checklist

| **Section and Topic** | **Item #** | **Checklist item** | **Location where item is reported** |
| --- | --- | --- | --- |
| **TITLE** | | |  |
| Title | 1 | The report is identified as a systematic review. | Title |
| **ABSTRACT** | | |  |
| Abstract | 2 | See the PRISMA 2020 for Abstracts checklist. | Abstract |
| **INTRODUCTION** | | |  |
| Rationale | 3 | Describe the rationale for the review in the context of existing knowledge. | Introduction |
| Objectives | 4 | Provide an explicit statement of the objective(s) or question(s) the review addresses. | Introduction |
| **METHODS** | | |  |
| Eligibility criteria | 5 | Specify the inclusion and exclusion criteria for the review and how studies were grouped for the syntheses. | Section 2.1 |
| Information sources | 6 | Specify all databases, registers, websites, organisations, reference lists and other sources searched or consulted to identify studies. Specify the date when each source was last searched or consulted. | Section 2.1 |
| Search strategy | 7 | Present the full search strategies for all databases, registers and websites, including any filters and limits used. | Section 2.1 & Supplementary Table 3 |
| Selection process | 8 | Specify the methods used to decide whether a study met the inclusion criteria of the review, including how many reviewers screened each record and each report retrieved, whether they worked independently, and if applicable, details of automation tools used in the process. | Section 2.1 |
| Data collection process | 9 | Specify the methods used to collect data from reports, including how many reviewers collected data from each report, whether they worked independently, any processes for obtaining or confirming data from study investigators, and if applicable, details of automation tools used in the process. | Section 2.1 |
| Data items | 10a | List and define all outcomes for which data were sought. Specify whether all results that were compatible with each outcome domain in each study were sought (e.g. for all measures, time points, analyses), and if not, the methods used to decide which results to collect. | Section 2.1 |
|  | 10b | List and define all other variables for which data were sought (e.g. participant and intervention characteristics, funding sources). Describe any assumptions made about any missing or unclear information. | Section 2.1 |
| Study risk of bias assessment | 11 | Specify the methods used to assess risk of bias in the included studies, including details of the tool(s) used, how many reviewers assessed each study and whether they worked independently, and if applicable, details of automation tools used in the process. | Section 2.1 & Section 2.4 & Section 2.5 |
| Effect measures | 12 | Specify for each outcome the effect measure(s) (e.g. risk ratio, mean difference) used in the synthesis or presentation of results. | - |
| Synthesis methods | 13a | Describe the processes used to decide which studies were eligible for each synthesis (e.g. tabulating the study intervention characteristics and comparing against the planned groups for each synthesis (item #5)). | Section 2.1 |
|  | 13b | Describe any methods required to prepare the data for presentation or synthesis, such as handling of missing summary statistics, or data conversions. | Section 2.2 & Section 2.3 |
|  | 13c | Describe any methods used to tabulate or visually display results of individual studies and syntheses. | Section 2.2 ~ Section 2.5 |
|  | 13d | Describe any methods used to synthesize results and provide a rationale for the choice(s). If meta-analysis was performed, describe the model(s), method(s) to identify the presence and extent of statistical heterogeneity, and software package(s) used. | Section 2.2 ~ Section 2.5 |
|  | 13e | Describe any methods used to explore possible causes of heterogeneity among study results (e.g. subgroup analysis, meta-regression). | Section 2.4 |
|  | 13f | Describe any sensitivity analyses conducted to assess robustness of the synthesized results. | Section 2.4 |
| Reporting bias assessment | 14 | Describe any methods used to assess risk of bias due to missing results in a synthesis (arising from reporting biases). | Section 2.5 |
| Certainty assessment | 15 | Describe any methods used to assess certainty (or confidence) in the body of evidence for an outcome. | - |
| **RESULTS** | | |  |
| Study selection | 16a | Describe the results of the search and selection process, from the number of records identified in the search to the number of studies included in the review, ideally using a flow diagram. | Section 3.1 & Figure 1 |
|  | 16b | Cite studies that might appear to meet the inclusion criteria, but which were excluded, and explain why they were excluded. | Figure 1 |
| Study characteristics | 17 | Cite each included study and present its characteristics. | Section 3.1 |
| Risk of bias in studies | 18 | Present assessments of risk of bias for each included study. | Table 1 |
| Results of individual studies | 19 | For all outcomes, present, for each study: (a) summary statistics for each group (where appropriate) and (b) an effect estimate and its precision (e.g. confidence/credible interval), ideally using structured tables or plots. | Section 3.1 & Table 1 |
| Results of syntheses | 20a | For each synthesis, briefly summarise the characteristics and risk of bias among contributing studies. | Section 3.1~ Section 3.5 |
|  | 20b | Present results of all statistical syntheses conducted. If meta-analysis was done, present for each the summary estimate and its precision (e.g. confidence/credible interval) and measures of statistical heterogeneity. If comparing groups, describe the direction of the effect. | Section 3.1~ Section 3.5 |
|  | 20c | Present results of all investigations of possible causes of heterogeneity among study results. | Section 3.5 |
|  | 20d | Present results of all sensitivity analyses conducted to assess the robustness of the synthesized results. | Section 3.4 |
| Reporting biases | 21 | Present assessments of risk of bias due to missing results (arising from reporting biases) for each synthesis assessed. | Section 3.5 |
| Certainty of evidence | 22 | Present assessments of certainty (or confidence) in the body of evidence for each outcome assessed. | - |
| **DISCUSSION** | | |  |
| Discussion | 23a | Provide a general interpretation of the results in the context of other evidence. | Discussion |
|  | 23b | Discuss any limitations of the evidence included in the review. | Conclusion |
|  | 23c | Discuss any limitations of the review processes used. | Conclusion |
|  | 23d | Discuss implications of the results for practice, policy, and future research. | Conclusion |
| **OTHER INFORMATION** | | |  |
| Registration and protocol | 24a | Provide registration information for the review, including register name and registration number, or state that the review was not registered. | Section 2.1 |
|  | 24b | Indicate where the review protocol can be accessed, or state that a protocol was not prepared. | Section 2.1 |
|  | 24c | Describe and explain any amendments to information provided at registration or in the protocol. | - |
| Support | 25 | Describe sources of financial or non-financial support for the review, and the role of the funders or sponsors in the review. | Funding Sources |
| Competing interests | 26 | Declare any competing interests of review authors. | Declarations of Interest |
| Availability of data, code and other materials | 27 | Report which of the following are publicly available and where they can be found: template data collection forms; data extracted from included studies; data used for all analyses; analytic code; any other materials used in the review. | Data Availability Statement |

From: Page MJ, McKenzie JE, Bossuyt PM, Boutron I, Hoffmann TC, Mulrow CD, et al. The PRISMA 2020 statement: an updated guideline for reporting systematic reviews. BMJ 2021;372:n71. doi: 10.1136/bmj.n71 For more information, visit: <http://www.prisma-statement.org/>

**Table S2** PRIMSA Abstract Checklist

| **Topic** | **No.** | **Item** | **Reported?** |
| --- | --- | --- | --- |
| **TITLE** |  |  |  |
| **Title** | 1 | Identify the report as a systematic review. | Yes |
| **BACKGROUND** |  |  |  |
| **Objectives** | 2 | Provide an explicit statement of the main objective(s) or question(s) the review addresses. | Yes |
| **METHODS** |  |  |  |
| **Eligibility criteria** | 3 | Specify the inclusion and exclusion criteria for the review. | Yes |
| **Information sources** | 4 | Specify the information sources (e.g. databases, registers) used to identify studies and the date when each was last searched. | Yes |
| **Risk of bias** | 5 | Specify the methods used to assess risk of bias in the included studies. | Yes |
| **Synthesis of results** | 6 | Specify the methods used to present and synthesize results. | Yes |
| **RESULTS** |  |  |  |
| **Included studies** | 7 | Give the total number of included studies and participants and summarise relevant characteristics of studies. | Yes |
| **Synthesis of results** | 8 | Present results for main outcomes, preferably indicating the number of included studies and participants for each. If meta-analysis was done, report the summary estimate and confidence/credible interval. If comparing groups, indicate the direction of the effect (i.e. which group is favoured). | Yes |
| **DISCUSSION** |  |  |  |
| **Limitations of evidence** | 9 | Provide a brief summary of the limitations of the evidence included in the review (e.g. study risk of bias, inconsistency and imprecision). | Yes |
| **Interpretation** | 10 | Provide a general interpretation of the results and important implications. | Yes |
| **OTHER** |  |  |  |
| **Funding** | 11 | Specify the primary source of funding for the review. | Yes |
| **Registration** | 12 | Provide the register name and registration number. | Yes |

From: Page MJ, McKenzie JE, Bossuyt PM, Boutron I, Hoffmann TC, Mulrow CD, et al. The PRISMA 2020 statement: an updated guideline for reporting systematic reviews. MetaArXiv. 2020, September 14. DOI: 10.31222/osf.io/v7gm2. For more information, visit: www.prisma-statement.org

**Table S3** Search strategies for each database

| Databases | Search strategy |
| --- | --- |
| Embase | Search for rs-fMRI studies: ('cervical spondylosis'/exp OR 'cervical spondylosis' OR 'cs'/exp OR 'cs' OR 'csd' OR 'cervical spondylotic' OR 'cervical radiculopathy'/exp OR 'cervical radiculopathy' OR 'csr' OR 'cervical myelopathy'/exp OR 'cervical myelopathy' OR 'csm' OR 'csa'/exp OR 'csa' OR 'scs' OR 'dcm') AND ('reho' OR 'regional homogeneity'/exp OR 'regional homogeneity' OR 'amplitude of low-frequency fluctuations' OR 'alff' OR 'fractional amplitude of low-frequency fluctuations' OR 'falff') |
|  | Search for VBM studies: ('cervical spondylosis'/exp OR 'cervical spondylosis' OR 'cervical spondylotic' OR 'cervical radiculopathy'/exp OR 'cervical radiculopathy' OR 'cervical myelopathy'/exp OR 'cervical myelopathy' OR 'cs'/exp OR 'cs' OR 'csd' OR 'csm' OR 'csa'/exp OR 'csa' OR 'scs' OR 'dcm') AND ('voxel-based morphometry'/exp OR 'voxel-based morphometry' OR 'vbm' OR 'voxel-wise' OR 'voxel-based' OR 'volumetric' OR 'morphometry'/exp OR 'morphometry' OR 'gray matter'/exp OR 'gray matter') AND ('magnetic resonance imaging'/exp OR 'magnetic resonance imaging' OR 'mri'/exp OR 'mri' OR 'neuroimaging'/exp OR 'neuroimaging') |
|  | Search for SBM studies: ('cervical spondylosis'/exp OR 'cervical spondylosis' OR 'cervical spondylotic' OR 'cervical myelopathy'/exp OR 'cervical myelopathy' OR 'cervical radiculopathy'/exp OR 'cervical radiculopathy') AND ('sbm' OR 'surface-based morphometry' OR 'cortical thickness'/exp OR 'cortical thickness' OR 'surface area'/exp OR 'surface area' OR 'sulcus depth'/exp OR 'sulcus depth' OR 'gyrification index'/exp OR 'gyrification index' OR 'fractal dimension'/exp OR 'fractal dimension') |
|  | Search for DTI studies: ('cervical spondylosis'/exp OR 'cervical spondylosis' OR 'cervical spondylotic' OR 'cervical myelopathy'/exp OR 'cervical myelopathy' OR 'cervical radiculopathy'/exp OR 'cervical radiculopathy') AND ('dti' OR 'diffusion tensor imaging'/exp OR 'diffusion tensor imaging' OR 'diffusion tensor magnetic resonance imaging'/exp OR 'diffusion tensor magnetic resonance imaging' OR 'diffusion tensor mri'/exp OR 'diffusion tensor mri' OR 'diffusion tensor mris' OR 'diffusion tractography'/exp OR 'diffusion tractography') AND ('brain'/exp OR 'brain' OR 'cerebral' OR 'cortex'/exp OR 'cortex' OR 'subcortex'/exp OR 'subcortex' OR 'cortical' OR 'subcortical' OR 'cerebrum'/exp OR 'cerebrum') |
| Pubmed | Search for rs-fMRI studies: ("cervical spondylosis"[All Fields] OR "CS"[All Fields] OR "CSD"[All Fields] OR "cervical spondylotic"[All Fields] OR "cervical radiculopathy"[All Fields] OR "CSR"[All Fields] OR "cervical myelopathy"[All Fields] OR "CSM"[All Fields] OR "CSA"[All Fields] OR "SCS"[All Fields] OR "DCM"[All Fields]) AND ("ReHo"[All Fields] OR "regional homogeneity"[All Fields] OR "amplitude of low-frequency fluctuations"[All Fields] OR "ALFF"[All Fields] OR "fractional amplitude of low-frequency fluctuations"[All Fields] OR "fALFF"[All Fields]) |
|  | Search for VBM studies: ("cervical spondylosis"[All Fields] OR "cervical spondylotic"[All Fields] OR "cervical radiculopathy"[All Fields] OR "cervical myelopathy"[All Fields] OR "CS"[All Fields] OR "CSD"[All Fields] OR "CSM"[All Fields] OR "CSA"[All Fields] OR "SCS"[All Fields] OR "DCM"[All Fields]) AND ("voxel-based morphometry"[All Fields] OR "VBM"[All Fields] OR "voxel-wise"[All Fields] OR "voxel-based"[All Fields] OR "volumetric"[All Fields] OR "morphometry"[All Fields] OR "gray matter"[All Fields]) AND ("magnetic resonance imaging"[All Fields] OR "MRI"[All Fields] OR "neuroimaging"[All Fields]) |
|  | Search for DTI studies: ("cervical spondylosis"[All Fields] OR "cervical spondylotic"[All Fields] OR "cervical myelopathy"[All Fields] OR "cervical radiculopathy"[All Fields]) AND ("DTI"[All Fields] OR "diffusion tensor imaging"[All Fields] OR "diffusion tensor magnetic resonance imaging"[All Fields] OR "diffusion tensor MRI"[All Fields] OR "diffusion tensor MRIs"[All Fields] OR "Diffusion Tractography"[All Fields]) AND ("brain"[All Fields] OR "cerebral"[All Fields] OR "cortex"[All Fields] OR "subcortex"[All Fields] OR "cortical"[All Fields] OR "subcortical"[All Fields] OR "cerebrum"[All Fields]) |
|  | Search for SBM studies: ("cervical spondylosis"[All Fields] OR "cervical spondylotic"[All Fields] OR "cervical myelopathy"[All Fields] OR "cervical radiculopathy"[All Fields]) AND ("SBM"[All Fields] OR "surface-based morphometry"[All Fields] OR "cortical thickness"[All Fields] OR "surface area"[All Fields] OR "sulcus depth"[All Fields] OR "gyrification index"[All Fields] OR "fractal dimension"[All Fields]) |
| Web of Science | Search for rs-fMRI studies: (TS=(“cervical spondylosis” OR “CS” OR “CSD” OR “cervical spondylotic” OR “cervical radiculopathy” OR “CSR” OR “cervical myelopathy” OR “CSM” OR “CSA” OR “SCS” OR “DCM”)) AND TS=(“ReHo” OR “regional homogeneity” OR “amplitude of low-frequency fluctuations” OR “ALFF” OR “fractional amplitude of low-frequency fluctuations” OR “fALFF”) |
|  | Search for VBM studies: ((TS=(“cervical spondylosis” OR “cervical spondylotic” OR “cervical radiculopathy” OR “cervical myelopathy” OR “CS” OR “CSD” OR “CSM” OR “CSA” OR “SCS” OR “DCM”)) AND TS=(“voxel-based morphometry” OR “VBM” OR “voxel-wise” OR “voxel-based” OR “volumetric” OR “morphometry” OR “gray matter”)) AND TS=(“magnetic resonance imaging” OR “MRI” OR “neuroimaging”) |
|  | Search for SBM studies: (TS=(“cervical spondylosis” OR “cervical spondylotic” OR “cervical myelopathy” OR “cervical radiculopathy”)) AND TS=(“SBM” OR “surface-based morphometry” OR “cortical thickness” OR “surface area” OR “sulcus depth” OR “gyrification index” OR “fractal dimension”) |
|  | Search for DTI studies: ((ALL=(“cervical spondylosis” OR “cervical spondylotic” OR “cervical myelopathy” OR “cervical radiculopathy”)) AND ALL=(“DTI” OR “diffusion tensor imaging” OR “diffusion tensor magnetic resonance imaging” OR “diffusion tensor MRI” OR “diffusion tensor MRIs” OR “Diffusion Tractography”)) AND ALL=(“brain” OR “cerebral” OR “cortex” OR “subcortex” OR “cortical” OR “subcortical” OR “cerebrum”) |
| CNKI | Search for rs-fMRI studies: (“颈椎病”) AND (“局部一致性” OR “低频振幅” OR “比率低频振幅” OR “静息态功能磁共振”) |
|  | Search for VBM studies: (“颈椎病”) AND (“形态学” OR “灰质体积” OR “神经成像” OR “磁共振成像”) |
|  | Search for SBM studies: (“颈椎病”) AND (“基于表面的形态测量” OR “表面积” OR “皮层厚度” OR “沟深” OR “皮层褶皱” OR “皮层复杂度”) |
|  | Search for DTI studies: (“颈椎病”) AND (“扩散张量成像” OR “弥散张量成像”) |
| Wangfang Data | Search for rs-fMRI studies: (“颈椎病”) AND (“局部一致性” OR “低频振幅” OR “比率低频振幅” OR “静息态功能磁共振”) |
|  | Search for VBM studies: (“颈椎病”) AND (“形态学” OR “灰质体积” OR “神经成像” OR “磁共振成像”) |
|  | Search for SBM studies: (“颈椎病”) AND (“基于表面的形态测量” OR “表面积” OR “皮层厚度” OR “沟深” OR “皮层褶皱” OR “皮层复杂度”) |
|  | Search for DTI studies: (“颈椎病”) AND (“扩散张量成像” OR “弥散张量成像”) |

**Table S4** Quality assessment

Criteria for objective assessment of methodological quality of individual studies

| **Category 1: Sample characteristics (10)** |
| --- |
| 1. Patients were evaluated with specific standardized diagnostic criteria (1) |
| 2. Important demographic data (age and gender) were reported with mean (or median) and standard deviations (or range)) (2) |
| 3. Healthy comparison subjects were evaluated to exclude psychiatric and medical illnesses and demographic data was reported (1) |
| 4. Important clinical variables (e.g. illness duration, medication status, HAMA scores, HAMD scores) were reported with mean (or median) and standard deviations (or range)) (4) |
| 5. Sample size per group > 10 (2) |
| **Category 2: Methodology and reporting (10)** |
| 1. Whole brain analysis was automated with no a-priori regional selection (3) |
| 2. Magnet strength at least 1.5T (1) |
| 3. At least 5 minutes of resting state acquisition (1) |
| 4. Whole brain coverage of resting scans (1) |
| 5. The acquisition and preprocessing techniques were clearly described so that they could be reproduced (1) |
| 6. Coordinates reported in a standard space (1) |
| 7. Significant results are reported after correction for multiple testing using a standard statistical procedure (FDR, FWE or permutation-based methods) (1) |
| 8. Conclusions were consistent with the results obtained and the limitations were discussed (1) |

A maximum score of 20 for each study, allocated as per the criteria specified above.

**Table S5** The intersection results of multimodal and unimodal meta-analytic results

| **Description** | **Peak MNI coordinates** | **Cluster size** | **Cluster breakdown (no. of voxels)** |
| --- | --- | --- | --- |
|  | **(x, y, z)** |  |  |
| **Intersection results of multimodal and VBM meta-analytic results** | | | |
| Temporal_Sup_R (aal) | 50,-24,10 | 650 | Temporal_Sup_R (429)  Rolandic_Oper_R (161)  SupraMarginal_R (36)  Heschl_R (19) |
| Paracentral_Lobule_R (aal) | 8,-32,66 | 55 | Paracentral_Lobule_R (52) |
| **Intersection results of multimodal and rs-fMRI meta-analytic results** | | | |
| Temporal_Sup_R (aal) | 60,-38,8 | 90 | Temporal_Sup_R (82) |
| Parietal_Inf_L (aal) | -48,-26,44 | 456 | Postcentral_L (254)  Parietal_Inf_L (158)  SupraMarginal_L (38) |

**Abbreviations**: MNI, Montreal Neurological Institute; VBM, voxel-based morphometry; rs-fMRI, resting-state functional magnetic resonance imaging; Temporal_Sup_R, right superior temporal gyrus; Rolandic_Oper_R, right rolandic operculum; SupraMarginal_R, right supramarginal gyrus; Heschl_R, right heschl gyrus; Paracentral_Lobule_R, right paracentral lobule; Temporal_Sup_R, right superior temporal gyrus; Parietal_Inf_L, left inferior parietal gyri; Postcentral_L, left postcentral gyrus; SupraMarginal_L, left supramarginal gyrus.

Regions with less than 10 voxels are not reported in the cluster breakdown.


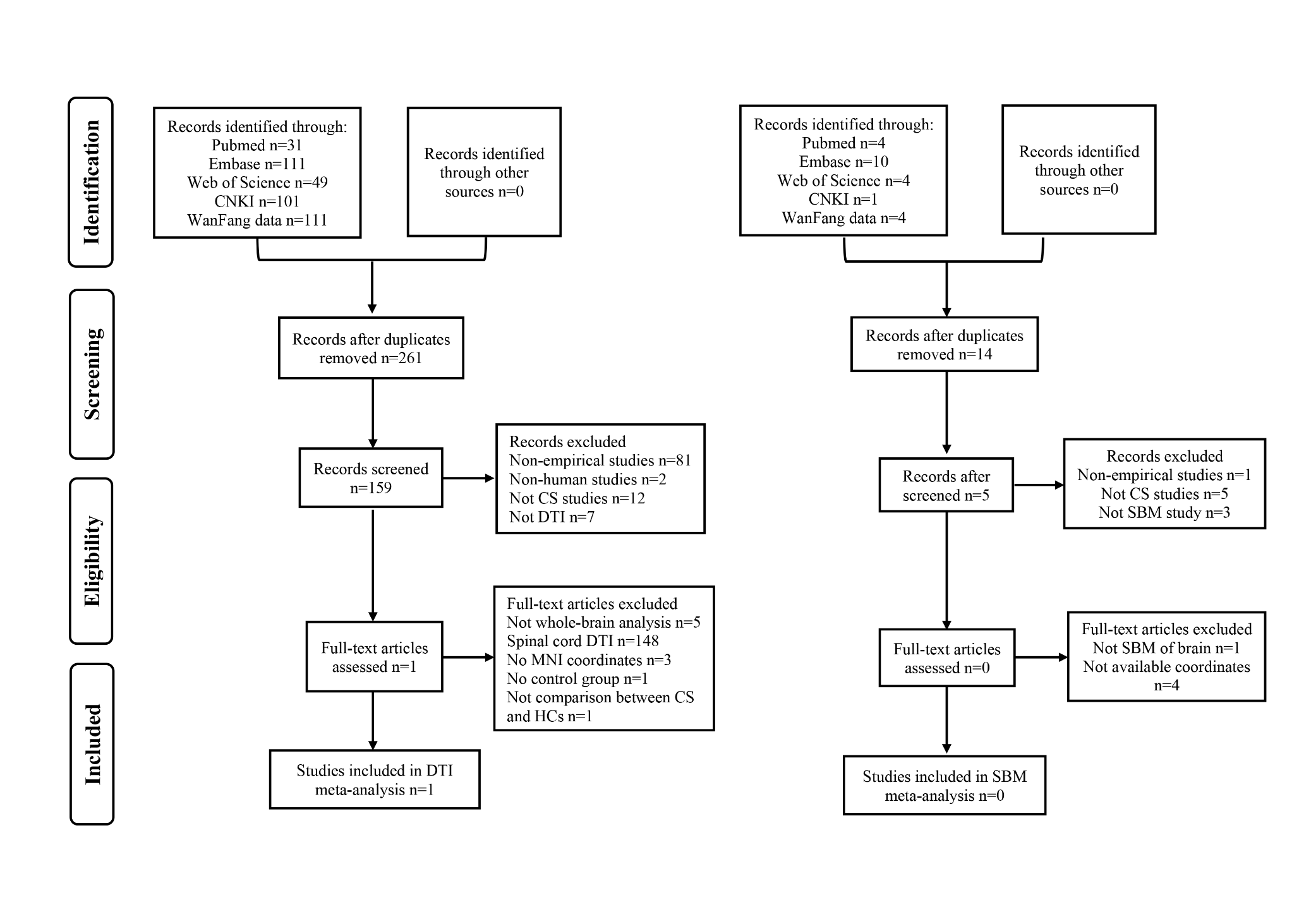


**Figure S1** Flow diagram of literature search and study selection

**Abbreviations**: CNKI, Chinese National Knowledge Infrastructure; CS, cervical spondylosis; HCs, healthy controls; DTI, diffusion tensor imaging; MNI, Montreal Neurological Institute; SBM, surface-based morphometry.


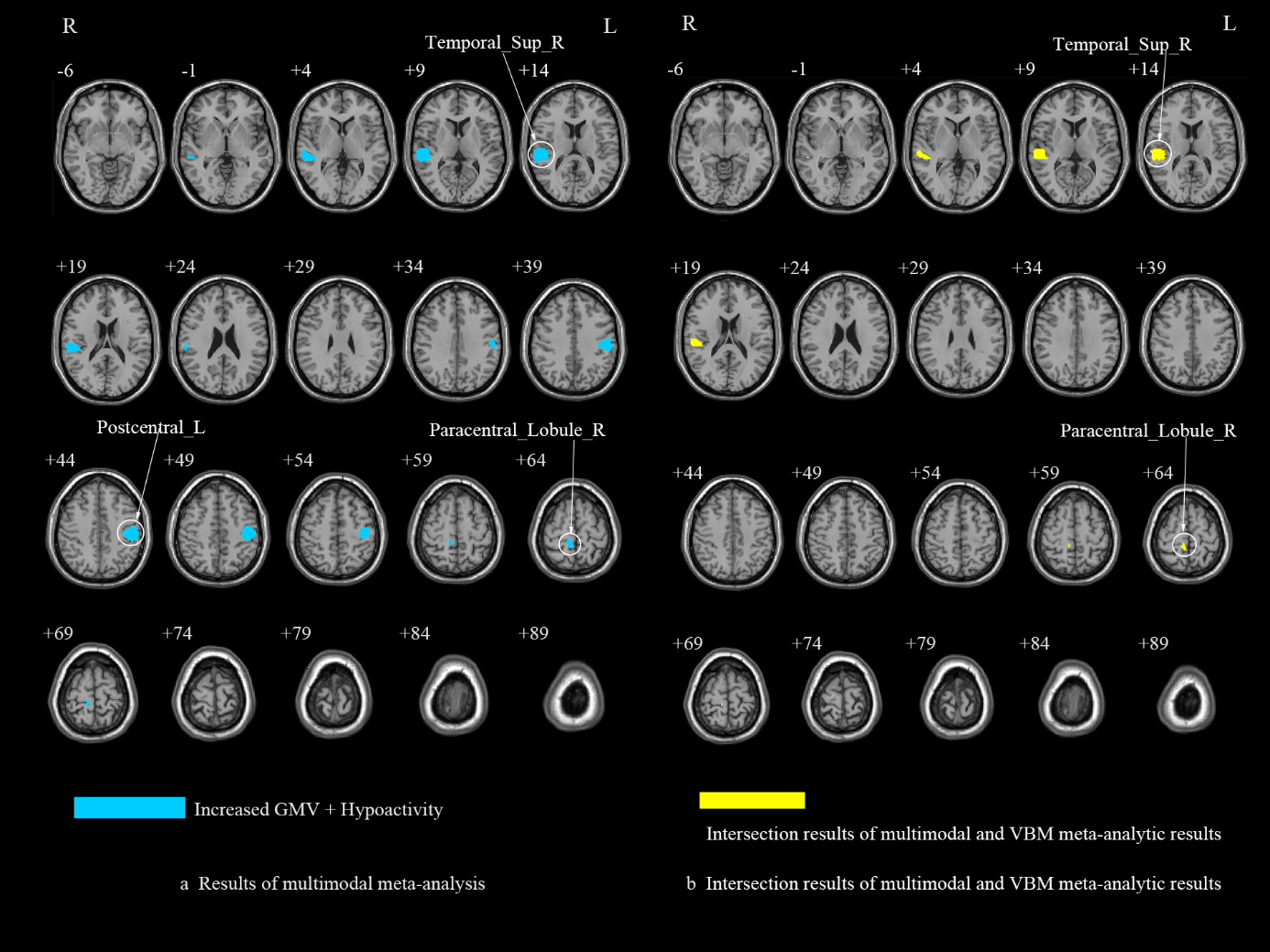


**Figure S2** (a). The results of multimodal meta-analysis; (b). The intersection results of multimodal and VBM meta-analytic results

**Abbreviations**: VBM, voxel-based morphometry; Temporal_Sup_R, right superior temporal gyrus; Postcentral_L, left postcentral gyrus; Paracentral_Lobule_R, right paracentral lobule; GMV, gray matter volume.


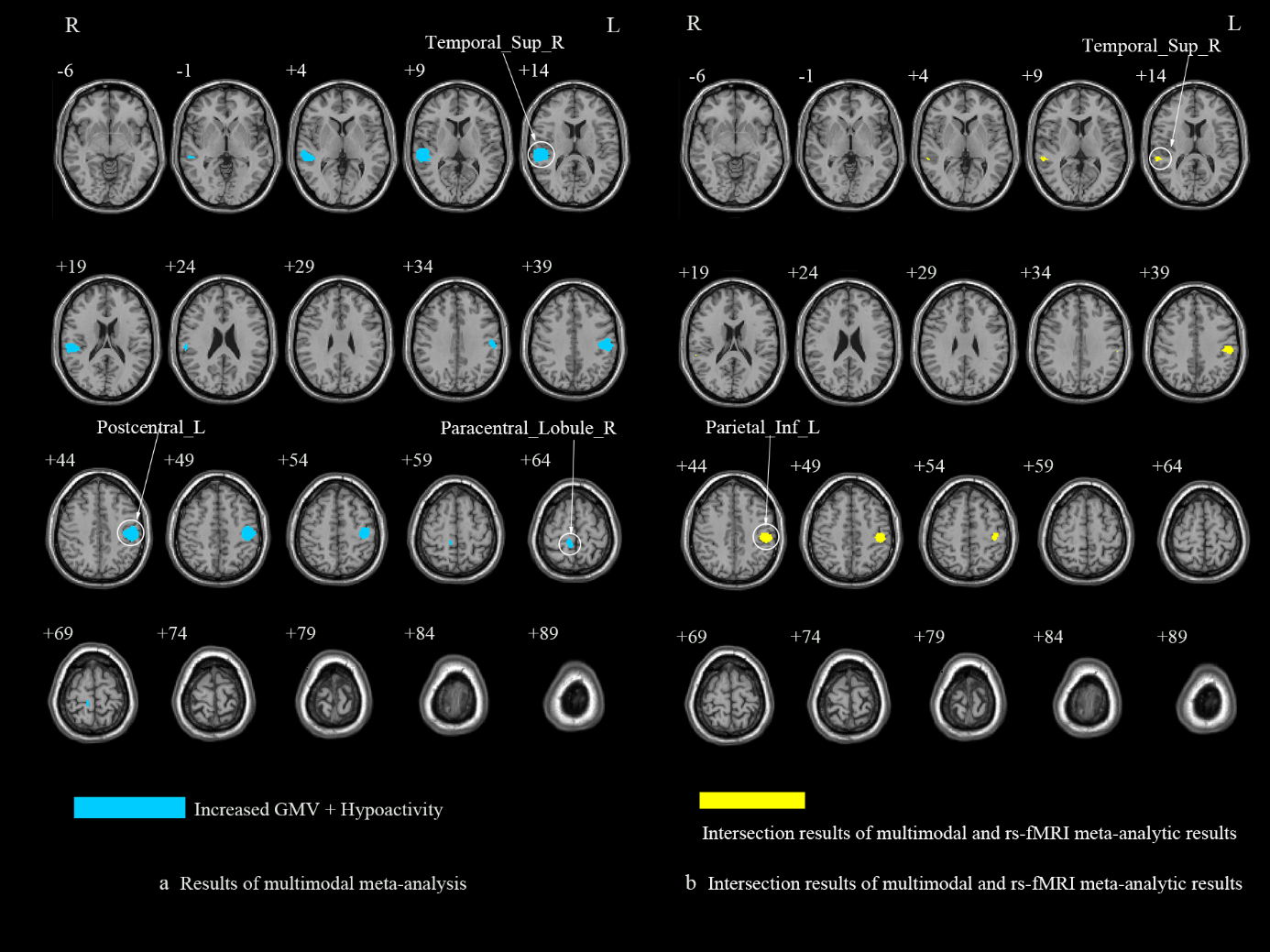


**Figure S3** (a). The results of multimodal meta-analysis; (b). The intersection results of multimodal and rs-fMRI meta-analytic results

**Annotation:** The Postcentral_L reported in the multimodal meta-analysis in Figure S2a was also included in the cluster (with 456 voxels) where the peak coordinate was located in the Parietal_Inf_L in Figure S2b, of which the Postcentral_L was the largest brain region with 254 voxels.

**Abbreviations**: rs-fMRI, resting-state functional magnetic resonance imaging; Temporal_Sup_R, right superior temporal gyrus; Postcentral_L, left postcentral gyrus; Paracentral_Lobule_R, right paracentral lobule; Parietal_Inf_L, left inferior parietal gyri; GMV, gray matter volume.
